# A novel multistep approach to standardize the reported risk factors for in-hospital falls: a proof-of-concept study

**DOI:** 10.3389/fpubh.2024.1390185

**Published:** 2024-06-12

**Authors:** Fabio La Porta, Giorgia Valpiani, Giada Lullini, Antonella Negro, Leonardo Pellicciari, Erika Bassi, Serena Caselli, Valentina Pecoraro, Erika Govoni

**Affiliations:** ^1^IRCCS Istituto delle Scienze Neurologiche di Bologna, Bologna, Italy; ^2^Research and Innovation Unit, Biostatistics and Clinical Trial Area, University Hospital of Ferrara, Ferrara, Italy; ^3^Innovation in Healthcare and Social Services, Emilia-Romagna Region, Bologna, Emilia-Romagna, Italy; ^4^Department of Translational Medicine, University of Eastern Piedmont, Novara, Italy; ^5^Unità Operativa Complessa di Medicina Riabilitativa, Azienda Ospedaliero-Universitaria di Modena, Modena, Italy; ^6^Department of Laboratory Medicine and Pathology, AUSL Modena, Modena, Italy; ^7^Unità Organizzativa Riabilitazione Ospedaliera, Dipartimento Assistenziale Tecnico e Riabilitativo, Ausl Bologna, Bologna, Italy

**Keywords:** risk factors, systematic review, meta-analysis, accidental falls, hospitals

## Abstract

**Background:**

Uncertainty and inconsistency in terminology regarding the risk factors (RFs) for in-hospital falls are present in the literature.

**Objective:**

(1) To perform a literature review to identify the fall RFs among hospitalized adults; (2) to link the found RFs to the corresponding categories of international health classifications to reduce the heterogeneity of their definitions; (3) to perform a meta-analysis on the risk categories to identify the significant RFs; (4) to refine the final list of significant categories to avoid redundancies.

**Methods:**

Four databases were investigated. We included observational studies assessing patients who had experienced in-hospital falls. Two independent reviewers performed the inclusion and extrapolation process and evaluated the methodological quality of the included studies. RFs were grouped into categories according to three health classifications (ICF, ICD-10, and ATC). Meta-analyses were performed to obtain an overall pooled odds ratio for each RF. Finally, protective RFs or redundant RFs across different classifications were excluded.

**Results:**

Thirty-six articles were included in the meta-analysis. One thousand one hundred and eleven RFs were identified; 616 were linked to ICF classification, 450 to ICD-10, and 260 to ATC. The meta-analyses and subsequent refinement of the categories yielded 53 significant RFs. Overall, the initial number of RFs was reduced by about 21 times.

**Conclusion:**

We identified 53 significant RF categories for in-hospital falls. These results provide proof of concept of the feasibility and validity of the proposed methodology. The list of significant RFs can be used as a template to build more accurate measurement instruments to predict in-hospital falls.

## 1 Introduction

Falls are a growing and under-recognized public health issue. A recent WHO report (1) estimated that 684,000 fatal falls occur each year, making it the second leading cause of unintentional injury death after road traffic injuries. Many factors, including aging populations and sedentary lifestyles, will likely increase global fall-related injury rates in the next decades. Falls in hospital settings are among the most common hospital-acquired conditions and contribute to morbidity, mortality, and healthcare costs (2). Fall rates among hospitalized adults show great global variability, ranging from 3 to 11 falls per 1,000 bed days (3, 4). In particular, the Agency for Healthcare Research and Quality reports an inpatient fall rate of 7.6 per 1,000 discharges or 227,000 falls in hospitals in the United States in 2017 (5). Around 25% of hospital falls are injurious, resulting in fractures, soft-tissue injuries, and fear of falling (6). Given the enormous individual, social, and healthcare costs, preventing in-hospital falls is a public health priority (7).

For effective prevention of in-hospital falls, the early identification of inpatients at risk of falling is crucial for any intervention to prevent in-hospital falls (8). For this reason, several fall-predicting tools have been developed to summarize the individual patient’s risk into one single number (9). However, a 2013 guideline from the National Institute of Health Care Excellence (NICE) reported that all the tools screened did not have sufficient sensitivity and specificity for accurate and reliable predictions, thus not recommending the use of these tools to predict the individual’s patient risk of falling (9). There may be several explanations for the low predictive accuracy of fall prediction tools. First, although many studies have been conducted to identify the causality of falls, a direct comparison of their results is difficult due to methodological issues, including retrospective designs, different study populations, and follow-up periods. Second, although older age and a history of past falls have been reported as the most important key predictors of future falls in older people (10), accidental falls are likely to result from a complex interaction of multiple risk factors. Indeed, in-hospital falls have been associated with demographic, physical, psychological, medical, socioeconomic, environmental, and behavioral factors (9, 11–13) that interact dynamically and can change over time and settings (14). Thus, the attempt to summarize a multidimensional construct’s complexity, such as the risk of falling into a single number, could be a misleading oversimplification leading to ineffective interventions (9). Third, more than 400 risk factors have been described (9). This plethora of risk factors suggests a lack of clarity, consistency, and consensus regarding risk factor definitions (15), as well as issues surrounding selecting risk factors to be included in a given tool.

On the other hand, using clear and consistent terminology for risk factors would likely reduce their number, thus making selecting the most relevant risk factors much easier. This, in turn, may lead to an improvement in the diagnostic accuracy of fall prediction tools. Indeed, a workable solution to overcome the inconsistencies in risk factor definitions could be to link them to standardized healthcare concepts, such as those provided by conceptual categories of international health classifications. In particular, the World Health Organization Family of International Classifications (WHO-FIC) (14) is a group of integrated classification systems to be used alone or jointly to establish a common language, allowing comparisons of data across countries’ healthcare services and providing a conceptual framework of information dimensions related to health and health management.

Thus, this study aimed to provide proof of concept that it may be feasible to significantly reduce the reported inconsistencies in the description of fall risk factors and, subsequently, their number by adopting the standard terminology provided by international health classifications. We planned to reach this overall aim in the following four steps: (1) to perform a literature review to identify the fall risk factors among hospitalized adults; (2) to link the found risk factors to the corresponding WHO Health Classifications’ categories to reduce the heterogeneity of their definitions; (3) to perform a meta-analysis on the risk categories identified in the previous step to identify the significant ones to reduce further the number of relevant risk factors for hospitalized falls (16); (4) to refine the final list of significant categories by removing redundancies to reduce the number of risk factors further.

## 2 Materials and methods

### 2.1 Literature review

#### 2.1.1 Search strategy

The search was performed on four electronic databases: PubMed, EMBASE, Scopus, and CINAHL. The search strategy adopted was similar across the databases. Particularly, it was developed using the following keywords: “accidental falls,” “falls,” “falls in hospitals,” “risk factors,” “fall risk factor,” “hospital,” “hospitalization,” “acute care,” and “adult” (see Supplementary Table S1 for the full search strings). Given the constraints of time and resources and the extensive volume of available literature on the topic, we decided to limit our search to studies on humans published in either English or Italian from January 2015 to March 2022. The search results were exported and compiled into a common reference database using a reference manager. References were then de-duplicated to derive a unique set of records.

#### 2.1.2 Criteria for considering studies for this review

Amongst the retrieved references, we included primary studies evaluating risk factors for falls according to the following inclusion criteria:

1. Population: patients admitted to the hospital, of both genders, aged over 16 years;
2. Intervention: not applicable;
3. Comparison: not applicable;
4. Outcome: one or more fall(s) during the hospitalization;
5. Method: observational study.

To ensure comprehensive coverage and capture all relevant researchers, we screened all the primary studies included in any secondary studies we retrieved, thus including them if they met the above inclusion criteria.

#### 2.1.3 Study selection and data extraction

Two investigators (EB and EG) independently examined the search results and screened the titles and abstracts to exclude irrelevant reports. A third reviewer (GV) solved any disagreement. The full text of the selected articles was retrieved and critically evaluated for eligibility, and their reference lists were manually scanned to identify further eligible studies. In the case of multiple publications from the same study, we selected the most updated one and extracted the data for the maximum possible length of follow-up.

From each of the included studies, the following data were extracted: author, study design, location, publication year, inclusion criteria, sample size, percentage of male/female, age, Odds Ratio (OR) or Relative Risk (RR) with its 95% Confidence Interval (95%CI). When the OR or the RR was not provided, we computed a crude OR if possible (17).

#### 2.1.4 Qualitative assessment of selected studies

Four independent reviewers (AN, SM, VP, and GV) evaluated the methodological quality of each included study. Studies were assessed using tailored National Institute of Health (NIH) Quality assessment tools for Case–control studies and for Observational cohort and cross-sectional studies (18).

The checklist included 12 and 14 questions, respectively, for case–control and cross-sectional studies. Possible answers included “yes,” “no,” “not reported,” “cannot determine,” or “not applicable.” Each study was rated for its overall quality as “good,” ‘fair’, or ‘poor’, based on the significance of the risk of bias and the consequent internal validity of its results. For instance, if a study had a ‘fatal flaw,’ the given rating was ‘poor’.

### 2.2 Linking of risk factors to international health classification categories

#### 2.2.1 Selection of health classifications

Fall risk factors are usually categorized into intrinsic (15) (medical conditions, multiple aspects of functioning, medications, personal factors) and extrinsic (environmental factors) factors. According to this initial conceptual framework categorization, within the WHO-FIC, we considered several classifications with a hierarchical structure (i.e., the more distal the category, the more detailed the health concept is). Thus, we considered the following classifications for linking:

- The International Classification of Functioning, Disability, and Health (ICF, 2017 version) that includes specific categories for functioning/disability (body functions, body structures, activity, and participation) as well as environmental and personal factors;
- The International Classification of Diseases version 10 (ICD-10), that includes specific categories for medical conditions (diagnoses);
- The Anatomical Therapeutic Chemical Classification (ATC) (19) for medications and drugs.

We excluded:

- the International Classification of Diseases version 11 (ICD-11), as it was not yet available at the time of data analysis;
- the International Classification of Nursing Practice (ICNP), given its polyhierarchical structure;
- The International Classification of Health Interventions (ICHI), as it only provides extension codes linked either to ICF or ICD-11.

#### 2.2.2 Linking risk factors to a health classification

The risk factors extracted from the literature review were then linked to three selected health classifications (ICF, ICD-10, and ATC), which were used as theoretical reference models. The linking was conducted by two clinicians (FLP, SC) who had almost 15 years of clinical and research experience in applying the ICF linking technique (20). They independently linked each extracted risk factor to one or more of the above classifications using a modified version of the standard linking techniques available for ICF (21). In particular, they employed an algorithm by which each risk factor was linked at least to one of the following definitions of health domains, which, in turn, identified the chosen classification:

- b (body function): the physiological functions of body systems, including psychological functions (ICF);
- s (body structure): anatomical parts of the body such as organs, limbs, and their components (ICF);
- d (activity and participation): the execution of a task or action by an individual or an involvement in a life situation (ICF);
- e (environmental factor): the physical, social, and attitudinal environment in which people live and conduct their lives (ICF);
- pf (person factor): the particular background of an individual’s life and living, and comprise features of the individual that are not part of a health condition or health states (e.g., gender, race, age, other health conditions, fitness, lifestyle, habits, upbringing, coping styles, social background, education, profession, past and current experience, overall behavior pattern and character style, individual psychological assets and other characteristics [ICF]);
- nd (not defined); any aspect of functioning, other than pf, not yet classified within the ICF;
- hc (health condition): a diagnosis or a health condition (ICD);
- dr (drug): any natural or human-made object or substance gathered, processed, or manufactured for medicinal purposes (ATC);
- nc (not classifiable): any risk factor not classifiable according to any of the previous labels.

According to this algorithm, each risk factor could be linked to one or more classifications. A third reviewer (GL) solved any disagreement.

#### 2.2.3 Linking risk factors to specific categories of health classifications

The next step was performed by three clinicians (FLP, SC, EG, and GL), who independently linked each risk factor to the appropriate categories of the identified classification. In particular, each risk factor was linked to the following hierarchical levels of each classification:

- For ICF: 1st-level categories (chapters), blocks, and 2nd-level categories;
- For ICD-10: chapters, blocks, categories, and subcategories;
- For ATC: 1st-and 2nd-level categories.

In case of discrepancy about the linking outcome for some specific risk factors, the four investigators resolved by consensus, choosing together the most appropriate categories according to indications given by the latest version of the ICF linking rules (21).

### 2.3 Meta-analysis

As all risk factors were traced back to specific categories of the three international classifications, it was possible to aggregate the single factors within the same category and then carry out the meta-analyses. We estimated a pooled OR (based on OR for case–control and cross-sectional studies and on RR or hazard risk for cohort studies) for each risk factor using random effect models (22). The pooled OR was derived from the inverse variance method, which involved computing the weighted average using standard error. All meta-analyses were performed using Stata software (version 14) (23).

The meta-analyses were carried out using a bottom-up approach. The latter entailed starting the meta-analysis from the more ‘distal categories’ and proceeding subsequently with the more ‘proximal categories.’ In doing so, if the estimated pooled OR was not significant for a given category, all the linked risk factors were excluded from the meta-analysis of the upper-level category.

### 2.4 Refinement of the final list of significant risk factors

The significant risk factors linked to the various health classifications were then screened independently by two clinicians (FLP, SC) looking for redundancies (i.e., the same risk factors linked to categories of different classifications). When redundancies were found, just one category was retained according to the following criteria: most appropriate classification (i.e., ICD-10 for medical diagnosis vs. ICF for aspects of functioning), higher pooled OR, and higher number of selected studies. Finally, categories with pooled OR < 1 (protective factors) were excluded. Any disagreement was solved by a third reviewer (GL).

## 3 Results

### 3.1 Literature review

#### 3.1.1 Study identification and selection

**Figure 1:**
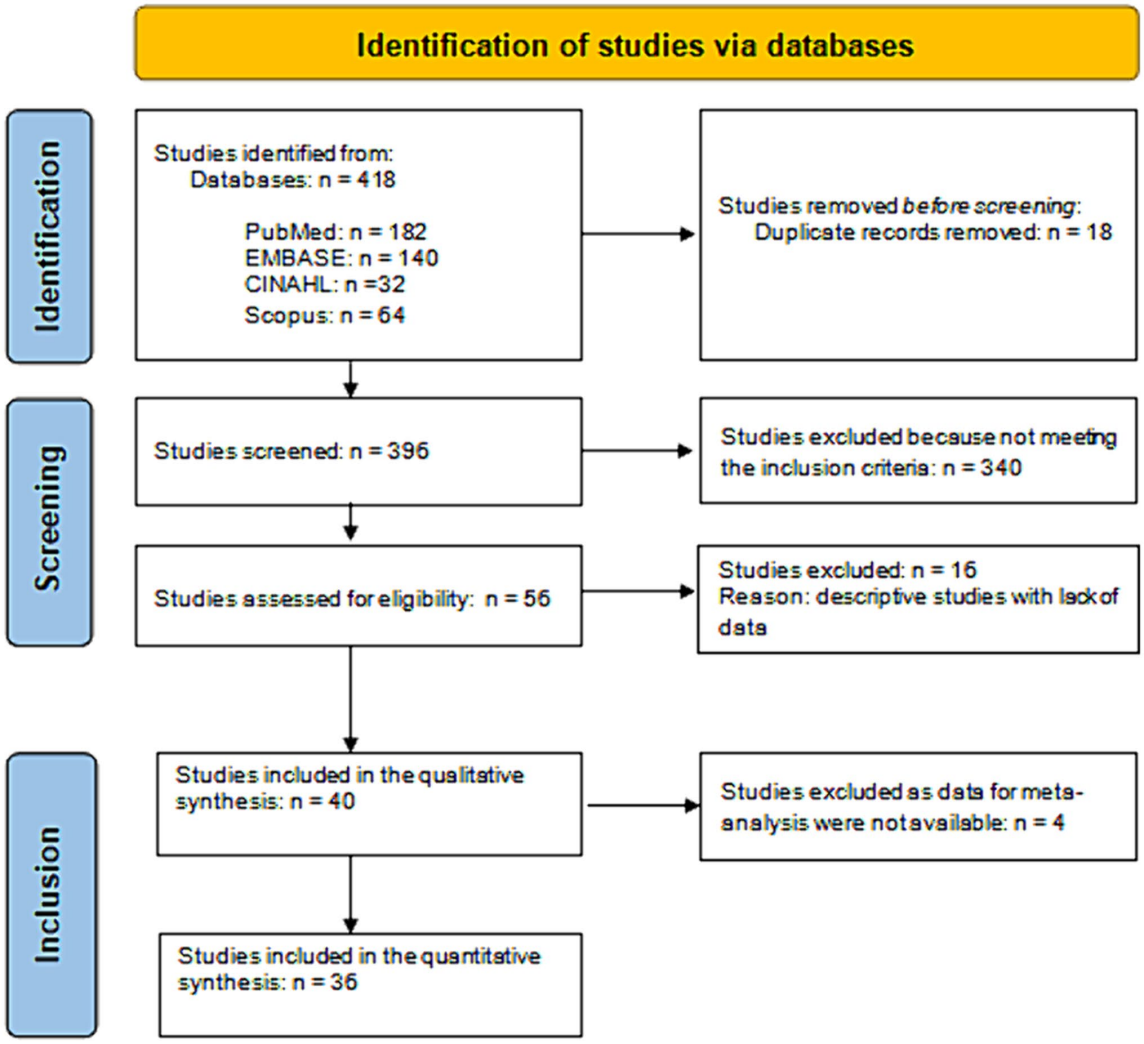
Flow diagram of the literature review.

After excluding duplicates and irrelevant records, the literature search identified 400 references (Figure 1). Of these, 344 were excluded because they did not meet the inclusion criteria. Thus, we found 56 studies eligible for inclusion and obtained full-text details. From full-text analysis, 16 further studies were excluded as descriptive studies with a lack of data. Therefore, 40 articles (24–63) were included in the qualitative synthesis, yielding 3,495,552 included patients.

#### 3.1.2 Characteristics of the included studies

Amongst the 40 included studies, 19 were case–control, 9 were cross-sectional, and 12 were cohort studies. Detailed characteristics of the included studies are reported in Table 1. Most studies were conducted in the United States and had a mean/median cohort age < 80 years, with a prevalence of male subjects between 22.1 and 71.7%.

**Table 1:** Characteristics of the included studies investigating risk factors for falls.

Study	Study design	Country	Publication year	Sample size	Inclusion criteria	Age (mean (SD)/median [Q1-Q3])	Male *n* (%)	Study quality
Akgün et al. 2022 (23)	Cross sectional	Netherland	2022	905	Patients with age > 65 years	Falls: 82.0 [78.0–87.0] No Falls: 81.0 [76.0–85.0]	437 (48.3%)	Fair
al Tehewy et al. 2015 (24)	Cross sectional	Egypt	2015	411	Patients with age > 60 years	67.6 (6.7)	224 (54.5%)	Good
Aranda-Gallardo et al. 2017 (25)	Cohort	Spain	2017	977	Patients with age > 16 years and length of stay longer than 48 h	65.6 (17.6)	518 (53.0%)	Good
Aryee et al. 2017 (26)	Case control	United Kingdom	2017	437	Adult patients	67.7 (NR)	246 (56.3%)	Fair
Brand & Sundararajan, 2010 (27)	Cohort	Australia	2010	3,345,415	Patients with age > 18 years and length of stay longer than 48 h	75.9 (NR)	1,392,936 (41.6%)	Fair
Chang et al. 2011 (29)	Case control	Taiwan	2011	330	Patients with aged ≥65 years	76.2 (NR)	200 (60.6%)	Good
Cho et al. 2020 (28)	Case control	Korea	2020	1788	Patients with age > 18 years	61.0 (NR)	985 (55.1%)	Good
Cox et al. 2017 (30)	Case control	United States	2017	856	Patients with age > 18 years and a diagnosis of a hematological malignancy	Falls: 64.7 (12.2) No Falls: 64.5 (14.0)	453 (52.9%)	Fair
Eglseer et al. 2020 (31)	Cross sectional	Australia	2020	3,702	Patients with age > 65 years	77.6 (7.6)	1,676 (45.3%)	Poor
Fehlberg et al. 2017 (32)	Case control	United States	2017	1888	Adult patients	63.1 (17.8)	866 (45.6%)	Good
Forrest & Chen, 2016 (33)	Cross sectional	United States	2016	2,524	Patients admitted to an inpatient rehabilitation unit.	64.3 (NR)	1,312 (52%)	Good
Guzzo et al. 2015 (34)	Case control	Italy	2015	152	Patients admitted to a general hospital	67 (NR)	109 (71.7%)	Good
Hanger et al. 2014 (35)	Cross sectional	New Zealand	2013	401	Older patients (65 years and older) With stroke	Falls: 79.1 [73.0–84.0] No Falls: 79.8 [74.0–85.0]	185 (46.1%)	Poor
Hauer et al. 2020 (36)	Cohort	Germany	2020	102	Patients diagnosed with dementia	82.8 (6.2)	21 (20.6%)	Fair
Hou et al. 2017 (37)	Cohort	Taiwan	2016	37,437	Patients with age > 18 years and length of stay longer than 24 h	56.2 (NR)	NR	Fair
Ishibashi et al. 2020 (38)	Case control	Japan	2020	1,620	Hospitalized patients who had their first fall	76.2 (NR)	770 (47.5%)	Fair
Ishikuro et al. 2017 (39)	Cross sectional	Japan	2017	1,362	Hospitalized patients	57.1 (NR)	637 (46.8%)	Good
Jung & Park, 2018 (40)	Case control	Korea	2018	15,440	Patients with age > 18 years	58.3 (NR)	8,331 (54%)	Good
Juraschek et al. 2019 (41)	Cohort	United States	2019	3,973	Participants without known coronary heart disease, heart failure, or stroke	75.7 (5.0)	1,510 (38%)	Good
Kim et al. 2019 (42)	Cohort	Korea	2019	60,049	Patients admitted to integrated care units	61.2 (17.2)	26,388 (43.9%)	Fair
Lackoff et al. 2020 (43)	Cohort	Australia	2020	1849	Patients with age > 18 years	67 (18.3)	1,043 (56.4%)	Good
Lucero et al. 2019 (44)	Case control	United States	2019	814	Patients with age > 18 years	57.8 (NR)	397 (49.0%)	Good
Magnuszewki et al. 2020 (45)	Cross sectional	Poland	2020	358	Patients, admitted for the first time to the department of geriatrics	82 [76–86]	79 (22.1%)	Fair
Mamun & Lim, 2009 (46)	Case control	Singapore	2010	598	Patients with age > 65 years	75.8 (n.r)	361 (60.4%)	Fair
Mazur et al. 2016 (47)	Cross sectional	Poland	2016	788	Geriatric patients	79.5	268 (34.0%)	Fair
Morishita et al. 2022 (48)	Case control	Japan	2022	508	Patients with age > 18 years	Falls: 67.5 (14.3) No Falls: 67.5 (14.3)	290 (57.1%)	Good
Najafpour et al. 2019 (49)	Case control	Iran	2019	1,326	Patients admitted to a general hospital	57.8 (NR)	708 (53.4%)	Poor
Nanda et al. 2011 (50)	Case control	United States	2011	564	Geriatric-psychiatric patients	80.3 (NR)	123 (54.7%)	Poor
Noh et al. 2021 (51)	Case control	Korea	2021	620	Patients with age > 55 years	Falls:73.7 (8.4) No Falls:73.6 (8.4)	365 (58.9%)	Good
Obayashi et al. 2013 (52)	Cohort	Japan	2013	3,683	Hospitalized patients	56.5 (20.2)	1965 (53.4%)	Good
Oneil et al. 2018 (53)	Case control	United States	2015	476	Hospitalized patients	59.5 (NR)	446 (49.3%)	Good
Pauley et al. 2006 (54)	Cohort	Canada	2006	1,267	Inpatient rehabilitation patients with diagnosis of amputation	66.7 (12.6)	849 (67%)	Fair
Severo et al. 2018 (55)	Case control	Brazil	2018	358	Patients with age > 18 years	58.9 (16.2)	204 (54%)	Fair
Sullivan & Harding, 2019 (56)	Cohort	Australia	2019	149	Patients with stroke in rehabilitation	75.6 (NR)	85 (57%)	Good
Swartzell et al. 2013 (57)	Cross sectional	United States	2013	107	Patients aged 65 to 85 years	75 (NR)	44 (41%)	Fair
Toye et al. 2019 (58)	Cohort	Australia	2019	397	Patients with age > 70 years	84.8 (7.2)	169 (42.6%)	Good
Vela et al. 2018 (59)	Case control	United States	2017	168	Patients with age > 18 years	Falls: 56.6 (13.3) No Falls: 53.6 (11.3)	93 (55.4%)	Fair
Wedmann et al. 2019 (60)	Case control	Germany	2019	962	Patients with age > 65 years	82 (NR)	396 (41.0%)	Fair
Yip et et al. 2016 (61)	Case control	Singapore	2016	421	Patients with age > 21 years	65.1 (NR)	249 (59.2%)	Fair
Yu et al. 2010 (62)	Cohort	Canada	2010	370	Patients undergoing lower limb amputation	Falls: 64.6 (16.2) No Falls: 65.0 (17.1)	NR	Good

SD, standard deviation; Q1, first quartile; Q3, third quartile; N, number, NR, not reported.

#### 3.1.3 Methodological quality of the included studies

As shown in Table 1, the overall methodological quality of cross-sectional and cohort studies was classified as good (*N* = 10), fair (*N* = 9), and poor (*N* = 2) quality. All included studies clearly defined the research question and the outcome measures. Twenty studies (95%) specified the population and confounding variables, 15 (71%) studies had at least 50% of eligible participants, 17 (85%) reported that the subjects were recruited from similar populations, and 8 (40%) provided a sample size justification. The exposure of interest was measured before the outcome in 18 (90%) studies and was defined in 17 (81%) studies. Twelve studies (57%) examined different levels of exposure, 4 (19%) reported a timeframe sufficient to determine an association between exposure and outcome, and 3 (14%) assessed the exposure more than once. The blinding of the outcome assessor was reported in only one study, and 15 (71%) studies reported a loss to follow-up by 20% or less (Supplementary Table S2).

The overall methodological quality of case–control studies was good (*N* = 9), fair (*N* = 8), and poor (*N* = 2). All studies clearly defined the research question. All studies except one reported that the control subjects were recruited from similar populations that gave rise to the cases and clearly described the case population. Sixteen (84%) studies clearly explained the study population and reported using concurrent controls, but only 4 provided a sample size justification. Potential confounding variables were measured in 16 (84%) studies; 14 (74%) studies reported definitions, inclusion and exclusion criteria, or processes used to identify cases and controls, and 8 (42%) studies specified the random selection of case and control. The exposure/risk was clearly defined in 12 (63%) studies and occurred before the development of the condition in 13 (68%) studies. The assessor of exposure/risk was blinded to the case or controls in only two studies (Supplementary Table S3).

### 3.2 Linking of risk factors to international health classification categories

#### 3.2.1 Linking risk factors to a health classification

The literature review yielded 1,111 different records. Table 2 shows that it was possible to link 896 records (80.6%) to one classification only, whereas the remaining 19.4% (*n* = 215) were linked to two classifications. The ICF was the classification with the highest number of uniquely linked records (401, 36.1%), followed by the ATC (260, 23.4%), and finally the ICD-10 (235, 21.1%). Besides, 215 records (19.4%) were linked to the ICF and ICD-10 classifications. ICF or ICD-10 shared no records with ATC (Table 2). As shown in Table 3, the ICF was the classification with the highest number of linked records (616), followed by ICD-10 (450) and ATC (260).

**Table 2:** Outcome of the risk factor linking to health classifications.

	***N***	**%**
Number of records	1,111	
Not classified	0	0.0%
Linked to one classification only	896	80.6%
Linked to two classifications	215	19.4%
**Distribution by classification**		
ICF only	401	36.1%
ICD10 only	235	21.1%
ATC only	260	23.4%
ICF and ICD10	215	19.4%
ICF and ATC	0	0.0%
ICD10 and ATC	0	0.0%

*N*, number; %, percentage; ICF, International Classification of Functioning, Disability, and Health; ICD-10, International Classification of Diseases; ATC, Anatomical Therapeutic Chemical Classification.

**Table 3:** Outcome of the risk factor linking to health classifications (detailed view by category level).

	*N* records	*N* categories	*N* 1st-level categories	*N* blocks	*N* 2nd-level categories	*N* 3rd-level categories
**ICF**						
N categories	616	105	15	18	63	9
N records per category						
Minimum value			2	1	1	1
Median			34	13	4	4
Maximum value			329	52	64	17
**ICD-10**						
N categories	450	131	17	57	57	-
N records per category						
Minimum value			1	1	1	-
Median			13	3	3	-
Maximum value			84	67	38	-
**ATC**						
N categories	260	82	9	-	41	32
N records per category						
Minimum value			3	-	1	1
Median			11	-	3	2
Maximum value			115	-	48	19

*N*, number; ICF, International Classification of Functioning, Disability, and Health; ICD-10, International Classification of Diseases; ATC, Anatomical Therapeutic Chemical Classification.

#### 3.2.2 Linking risk factors to specific categories of health classifications

As shown in Table 3, the 616 ICF records were linked to 105 categories, divided into 15 first-level, 18 blocks, 63 s-level, and nine third-level categories, yielding a median of 34, 13, 4, and 4 records. Following the linking procedure, it was possible to link the 450 ICD-10 records to 131 categories. In particular, we identified 17 first-level, 57 blocks, and 57 s-level categories, with a median of 13, 3, and 3 records. Finally, the 260 ATC records could be linked to 82 categories: nine were first-level, 41 were second-level, and 32 were third-level categories. The median values of linked records for the three types of categories were, respectively, 11, 3, and 2.

The ICD-10 was the classification with the highest number of linked categories (131), followed by ICF (105) and ATC (82). The linkage process allowed the grouping of the 1,111 initial records into 152 risk factors with an overall 7.3 reduction factor.

### 3.3 Meta-analysis

The number of articles included in the quantitative synthesis was 36, as four articles had to be excluded because they had no data usable for the meta-analysis (Figure 1). Table 4 shows the significant combined ORs for the risk factors linked to the ICF classification. In particular, the meta-analysis identified 52 risk factors as non-significant (Supplementary Table S4). On the other hand, 50 risk factors linked to 523 records could be identified as significant. Their OR values ranged from 0.022 (b280-b289 Pain) to 8.633 (d420 Transferring oneself).

**Table 4:** Significant risk factors linked to the International Classification of Functioning, Disability, and Health.

Risk factor				*N* records	*OR*	Lower 95% CI	Upper 95% CI	*p*-value
**b1 Mental Functions**				63	2.133	1.630	2.793	<0.001
	b110-b139 Global Mental Functions			36	1.978	1.602	2.441	<0.001
		b110 Consciousness functions		9	3.660	2.637	5.079	<0.001
		b114 Orientation functions		5	3.214	2.692	3.837	<0.001
		b117 Intellectual functions		17	3.984	1.867	8.499	<0.001
	b140-189 Specific Mental Functions			27	2.334	1.506	3.618	<0.001
		b140 Attention functions		1	3.981	1.345	11.784	0.013
		b147 Psychomotor functions		5	4.761	3.271	6.930	<0.001
		b164 Higher-level cognitive functions		8	2.570	1.668	3.961	<0.001
		b180 Experience of self and time functions		1	4.010	2.865	5.612	<0.001
**b2 Sensory functions and pain**				24	2.796	1.429	5.472	0.003
	b210-b229 Seeing and related functions			8	3.867	1.643	9.103	0.002
		b210 Seeing functions		8	3.867	1.643	9.103	0.002
	b230-b249 Hearing and vestibular function			14	3.391	2.040	5.634	<0.001
		b230 Hearing functions		4	2.744	1.524	4.940	0.001
		b240 Sensations associated with hearing and vestibular function		10	3.840	1.609	9.166	0.002
	b250-b279 Additional sensory functions			1	2.768	1.126	6.804	0.027
		b265 Touch function		1	2.768	1.126	6.804	0.027
	b280-b289 Pain			1	0.022	0.018	0.028	<0.001
		b280 Sensation of pain		1	0.022	0.018	0.028	<0.001
**b4 Functions of the cardiovascular, haematological, immunological, and respiratory systems**				14	1.264	1.149	1.390	<0.001
	b410-b429 Functions of the cardiovascular system			9	1.168	1.001	1.363	0.049
	b430-b439 Functions of the haematological ann immunological system			5	1.431	1.235	1.583	<0.001
		b430 Haematological system functions		2	1.366	1.063	1.756	0.015
		b435 Immunological system function		3	1.366	1.139	1.639	0.001
**b5 Functions of the digestive, metabolic and endocrine systems**				33	1.363	1.136	1.636	0.001
	b510-b539 Functions related to the digestive system			16	-	-	-	n.s.
		b525 Defecation functions		1	4.209	1.571	11.278	0.004
	b540-b559 Functions related to metabolism and endocrine system			17	1.532	1.258	1.867	<0.001
		b540 General metabolic functions		11	1.432	1.123	1.825	0.004
			b5401 Carbohydrate metabolism	11	1.432	1.123	1.825	0.004
		b545 Water, mineral and electrolyte balance functions		4	1.764	1.443	2.157	<0.001
			b5452 Electrolyte balance	4	1.764	1.443	2.157	<0.001
**b7 Neuromuscoloskeletal and movement-related functions**				20	2.005	1.506	2.670	<0.001
	b730-b749 Muscle functions			8	1.728	1.077	2.775	0.023
		b730 Muscle power functions		8	1.728	1.077	2.775	0.023
	b750-b789 Movement functions			12	2.195	1.641	2.935	<0.001
		b755 Involuntary movement reaction functions		8	1.960	1.413	2.720	<0.001
		b770 Gait pattern functions		4	3.184	1.674	6.053	<0.001
**d4 Mobility**				24	2.127	1.532	2.953	<0.001
	d410-d429 Changing and maintaining body position			3	3.186	1.039	9.767	0.043
		d420 Transferring oneself		1	8.633	4.449	16.751	<0.001
	d450-d459 Walking and moving			21	2.006	1.411	2.853	<0.001
		d450 Walking		21	2.006	1.411	2.853	<0.001
**d5 Self-care**				4	2.911	1.060	7.994	0.038
		d510 Washing oneself		1	6.159	3.381	11.217	<0.001
**e1 Products and technology**				41	-	-	-	n.s.
		e110 Products or substances for personal consumption		18	1.556	1.196	2.024	0.001
		e120 Products and technology for personal indoor and outdoor mobility and transportation		4	3.420	1.349	8.674	0.010
**nd Not Defined**				2	1.660	1.043	2.644	0.033
		Frailty		2	1.660	1.043	2.644	0.033

*N*, number; OR, odds ratio, CI, confidence interval, n.s., non-significant.

The combined ORs for the risk factors linked to the ICD-10 classification are reported in Table 5. Specifically, 101 risk factors were identified as non-significant (Supplementary Table S5). Fifty-one significant risk factors were reported for a total of 509 records; the pooled OR values ranged from 0.199 (M00-M99 Unspecified diseases of the musculoskeletal system and connective tissue) to 3.789 (Y74.1 Therapeutic (nonsurgical) and rehabilitative devices).

**Table 5:** Significant risk factors linked to the International Classification of Diseases 10.

Risk factor				*N* records	OR	Lower 95% CI	Upper 95% CI	*p*-value
**02 Neoplasms**				10	1.619	1.442	1.818	<0.001
	C00-C97 Malignant neoplasms			10	1.619	1.442	1.818	<0.001
		C76-C80 Malignant neoplasms of ill-defined, secondary and unspecified sites		7	1.581	1.430	1.748	<0.001
**03 Diseases of the blood and blood forming organs…**				3	1.366	1.100	1.695	0.005
	D60-D64 Aplastic and other anaemias			3	1.366	1.100	1.695	0.005
		D64 Other anaemias		3	1.366	1.100	1.695	0.005
**04 Endocrine, nutritional and metabolic diseases**				30	1.336	1.109	1.608	0.002
	E10-E14 Diabetes mellitus			11	1.432	1.123	1.825	0.004
		E14 Unspecified diabetes mellitus		11	1.432	1.123	1.825	0.004
	E70-E90 Metabolic disorders			4	1.764	1.443	2.157	<0.001
		E86 Volume depletion		1	1.921	1.370	2.700	0.0002
		E87 Other disorders of fluid, electrolyte and acid–base balance		3	1.747	1.312	2.326	<0.001
**05 Mental and Behavioral disorders**				63	2.079	1.583	2.731	<0.001
	F00-F09 Organic, includic symptomatic, mental disorders			50	2.336	1.724	3.165	<0.001
		F00 Dementia in Alzheimer disease		1	0.242	0.134	0.438	<0.001
		F05 Delirium, not induced by alcohol and other psychoactive substances		26	3.310	2.616	4.186	<0.001
		F06 Other mental disorders due to brain damage and dysfunction and to physical disease		10	2.080	1.008	4.292	0.047
**06 Diseases of the nervous system**				17	1.499	1.043	2.153	0.029
	G20-G26 Extrapyramidal and movement disorders			7	2.647	1.502	4.666	0.001
		G20 Parkinson’s disease		7	2.647	1.502	4.666	0.001
	G40-G47 Episodic and paroxysmal disorders			4	1.759	1.013	3.053	0.045
		G40 Epilepsy		1	2.785	1.851	4.190	<0.001
	G80-G83 Cerebral palsy and other paralytic syndromes			1	3.106	2.952	3.268	<0.001
		G82 Paraplegia and tetraplegia		1	3.106	2.952	3.268	<0.001
**09 Disease of the circulatory system**				40	1.211	1.024	1.432	0.025
	I30-I52 Other forms of heart disease			10	1.343	1.046	1.723	0.021
		I50 Heart failure		9	1.384	1.071	1.789	0.013
	I70-I79 Diseases of arteries, arterioles and capillaries			3	1.501	1.351	1.666	<0.001
		I70 Atherosclerosis		3	1.501	1.351	1.666	<0.001
	K70-K77 Diseases of the liver			2	1.943	1.752	2.156	<0.001
		K76 Other diseases of the liver		2	1.943	1.752	2.156	<0.001
	K00-K93 Unspecified disease of the digestive system			1	0.541	0.343	0.852	0.003
	M05-M14 Inflammatory polyarthropaties			3	1.692	1.250	2.292	0.001
		M12 Other specific arthropaties		3	1.692	1.250	2.292	0.001
	M15-M19 Artrhosis			1	3.726	1.360	10.250	0.002
		M19 Other arthrosis		1	3.726	1.360	10.250	0.002
	M30-M36 Systemic connective tissue disorder			1	1.387	1.215	1.530	<0.001
		M35 Other systemic involvement of connective tissue		1	1.387	1.215	1.530	<0.001
	M00-M99 Unspecified diseases of the muscoloskeletal system and connective tissue			3	0.199	0.046	0.853	0.030
**18 Symptoms, signs, and abnormal clinical and laboratory findings**				33	2.040	1.684	2.470	<0.001
	R25-R29 Symptoms and signs involving the nervous and musculoskeletal systems			25	2.211	1.774	2.754	<0.001
		R29 Other symptoms and signs involving the nervous and musculoskeletal systems		25	2.211	1.774	2.754	<0.001
			R29.6 Tendency to fall, not elsewhere classified	25	2.211	1.774	2.754	<0.001
	R50-R69 General symptoms and signs			6	1.498	1.258	1.784	<0.001
		R55 Syncope and collapse		1	2.170	1.077	4.371	0.030
		R65 Systemic Inflammatory Response Syndrome		5	1.462	1.221	1.751	<0.001
**19 Injury, poisoning and certain other consequences of external causes**				10	1.304	1.021	1.666	0.034
	T08-T14 Injuries to unspecified part of trunk, limb or body region			5	1.390	1.048	1.844	0.022
**20 External causes of morbidity and mortality**				14	-	-	-	n.s.
	W00-X59 Other external causes of accidental injury			-	-	-	-	-
		W19 Unspecified Falls		1	2.650	1.313	5.350	0.007
	Y70-Y82 Medical devices associated with adverse incidents in diagnostic and therapeutic use			13	-	-	-	n.s.
		Y74 General hospital and personal-use devices associated with adverse incidents		13	-	-	-	n.s.
			Y74.1 Therapeutic (nonsurgical) and rehabilitative devices	3	3.789	1.359	10.560	0.011
**21 Factors influencing health status and contact with health services**				42	-	-	-	n.s.
	Z40-Z54 Persons encountering health services for specific procedures and health care			29	-	-	-	n.s.
		Z50 Care involving use of rehabilitation procedures		3	2.913	2.788	3.045	<0.001

*N*, number; OR, odds ratio, CI, confidence interval; n.s., non-significant. Note: for simplicity, the Roman numerals in ICD10 have been replaced by Arabic numerals.

Table 6 shows the combined ORs for the risk factors linked to the ATC classification. After excluding 65 risk factors because they were non-significant (Supplementary Table S6), the metanalyses yielded twenty-six significant risk factors for 294 records. The pooled OR values ranged from 0.348 (G04C Drugs used in benign prostatic hypertrophy) to 8.609 (B01AD Enzymes).

**Table 6:** Significant risk factors linked to the anatomical therapeutic chemical classification.

Risk factor	*N* records	OR	Lower 95% CI	Upper 95% CI	*p*-value
**A Alimentary tract and metabolism**	23	-	-	-	n.s.
A02 drugs for acid-related disorders	3	-	-	-	n.s.
A02A Antacids	1	0.690	0.501	0.950	0.023
A04 Antiemetics	3	0.691	0.557	0.858	0.001
A04A Antiemetics and antinauseants	1	0.610	0.450	0.827	0.001
A07 Antidiarrheal, intestinal antiinflammatory/antiinfective agents	2	1.874	1.324	2.651	<0.001
A10 Drugs used in diabetes	6	1.939	1.205	3.120	0.006
**B Blood and blood forming organs**	10	-	-	-	n.s.
B01 Antithrombotic agents	10	-	-	-	n.s.
B01AD Enzymes	1	8.609	3.486	21.264	<0.001
**C Cardiovascular system**	56	-	-	-	n.s.
C03 Diuretics	11	-	-	-	n.s.
C03D Potassium-sparing agents	2	0.556	0.375	0.827	0.004
**G Genito-urinary system and sex hormones**	3	-	-	-	n.s.
G04 Urologicals	2	-	-	-	n.s.
G04C Drugs used in benign prostatic hypertrophy	1	0.348	0.131	0.922	0.034
**L Antineoplastic and immunomodulating agents**	5	-	-	-	n.s.
L01 Antineoplastic agents	2	2.165	1.331	3.520	0.002
**M Muscolo-skeletal system**	7	-	-	-	n.s.
M03 Muscle relaxants	1	0.430	0.279	0.663	<0.001
**N Nervous System**	100	1.661	1.411	1.956	<0.001
N02 Analgesics	15	1.521	1.018	2.274	0.041
N02B Other analgesics and antipyretics	1	0.646	0.427	0.976	0.038
N03 Antiepileptics	12	2.009	1.508	2.677	<0.001
N05 Psycholeptics	44	1.829	1.566	2.138	<0.001
N05A Antipsychotic	17	1.862	1.439	2.411	<0.001
N05B Anxiolytics	12	1.930	1.572	2.369	<0.001
N05C Hypnotics and sedatives	15	1.685	1.193	2.380	0.003
N06 Psychoanaleptics	18	1.331	1.007	1.760	0.045
N06A Antidepressants	13	1.299	1.045	1.613	0.018
**R Respiratory system**	14	-	-	-	n.s.
R01 Nasal preparation	1	0.520	0.324	0.835	0.007
R03 Drugs for obustructive airway disease	6	0.790	0.662	0.943	0.009
R03B Other drugs for obstruct airway diseases, inhalants	2	0.660	0.511	0.851	0.001
R05 Cough and cold preparation	1	5.949	1.725	20.521	0.005

*N*, number; OR, odds ratio, CI, confidence interval; n.s., non-significant.

In summary, the meta-analyses allowed the initial risk factors to be reduced from 152 to 71, i.e., a 2.1 reduction factor.

### 3.4 Refinement of the final list of significant risk factors

Table 7 reported the combined ORs after deleting 18 categories because they were redundant or protective factors. In particular, eleven factors were excluded because they were protective (one, three, and seven were linked to ICF, ICF-10, and ATC), whereas seven (five from ICF and two from ICD-10) were excluded because they were deemed redundant.

**Table 7:** Refinement of the final list of significant risk factors.

CL	Proximal category	Intermediate category	Distal category	Notes	Rec	OR	L 95% CI	U 95% CI	*p*-value
ICF	b1 Mental functions	b110-b139 Global Mental Functions	b110 Consciousness functions	–	9	3.660	2.637	5.079	<0.001
ICF	b1 Mental functions	b110-b139 Global Mental Functions	b114 Orientation functions	–	5	3.214	2.692	3.837	<0.001
ICF	b1 Mental functions	b110-b139 Global Mental Functions	b117 Intellectual functions	–	17	3.984	1.867	8.499	<0.001
ICF	b1 Mental functions	b140-189 Specific Mental Functions	b140 Attention functions	–	1	3.981	1.345	11.784	0.013
ICF	b1 Mental functions	b140-189 Specific Mental Functions	b147 Psychomotor functions		5	4.761	3.271	6.930	<0.001
ICF	b1 Mental functions	b140-189 Specific Mental Functions	b164 Higher-level cognitive functions	–	8	2.570	1.668	3.961	<0.001
ICF	b1 Mental functions	b140-189 Specific Mental Functions	b180 Experience of self and time functions	–	1	4.010	2.865	5.612	<0.001
ICF	b2 Sensory functions and pain	b210-b229 Seeing and related functions	b210 Seeing functions	–	8	3.867	1.643	9.103	0.002
ICF	b2 Sensory functions and pain	b230-b249 Hearing and vestibular function	b230 Hearing functions	–	4	2.744	1.524	4.940	0.001
ICF	b2 Sensory functions and pain	b230-b249 Hearing and vestibular function	b240 Sensations associated with hearing and vestibular function	–	10	3.840	1.609	9.166	0.002
ICF	b2 Sensory functions and pain	b250-b279 Additional sensory functions	b265 Touch function	–	1	2.768	1.126	6.804	0.027
ICF	b5 Functions of the digestive, metabolic and endocrine systems	b510-b539 Functions related to the digestive system	b525 Defecation functions	–	1	4.209	1.571	11.278	0.004
ICF	b7 Neuromuscoloskeletal and movement-related functions	b730-b749 Muscle functions	b730 Muscle power functions	–	8	1.728	1.077	2.775	0.023
ICF	b7 Neuromuscoloskeletal and movement-related functions	b750-b789 Movement functions	b755 Involuntary movement reaction functions	–	8	1.960	1.413	2.720	<0.001
ICF	b7 Neuromuscoloskeletal and movement-related functions	b750-b789 Movement functions	b770 Gait pattern functions	–	4	3.184	1.674	6.053	<0.001
ICF	d4 Mobility	d410-d429 Changing and maintaining body position	d420 Transferring oneself	–	1	8.633	4.449	16.751	<0.001
ICF	d4 Mobility	d450-d459 Walking and moving	d450 Walking	–	21	2.006	1.411	2.853	<0.001
ICF	d5 Self-care	–	d510 Washing oneself	–	1	6.159	3.381	11.217	<0.001
ICF	e1 Products and technology	–	e110 Products or substances for personal consumption	Polypharmacotherapy	18	1.556	1.196	2.024	0.001
ICF	e1 Products and technology	–	e120 Products and technology for personal indoor and outdoor mobility and transportation	–	4	3.420	1.349	8.674	0.010
ICF	nd Not Defined	–	Frailty	–	2	1.660	1.043	2.644	0.033
ICD10	02 Neoplasms	C00-C97 Malignant neoplasms	C76-C80 Malignant neoplasms of ill-defined, secondary and unspecified sites	Malignant neoplasms	7	1.581	1.430	1.748	<0.001
ICD10	03 Diseases of the blood and blood forming organs…	D60-D64 Aplastic and other anaemias	D64 Other anaemias	Anemias	3	1.366	1.100	1.695	0.005
ICD10	04 Endocrine, nutritional and metabolic diseases	E10-E14 Diabetes mellitus	E14 Unspecified diabetes mellitus	Diabetes mellitus	11	1.432	1.123	1.825	0.004
ICD10	04 Endocrine, nutritional and metabolic diseases	E70-E90 Metabolic disorders	E86 Volume depletion	–	1	1.921	1.370	2.700	0.0002
ICD10	04 Endocrine, nutritional and metabolic diseases	E70-E90 Metabolic disorders	E87 Other disorders of fluid, electrolyte and acid–base balance	Hyponatriemia	3	1.747	1.312	2.326	<0.001
ICD10	06 Diseases of the nervous system	G20-G26 Extrapyramidal and movement disorders	G20 Parkinson’s disease	–	7	2.647	1.502	4.666	0.001
ICD10	06 Diseases of the nervous system	G40-G47 Episodic and paroxysmal disorders	G40 Epilepsy	–	1	2.785	1.851	4.190	<0.001
ICD10	06 Diseases of the nervous system	G80-G83 Cerebral palsy and other paralytic syndromes	G82 Paraplegia and tetraplegia	–	1	3.106	2.952	3.268	<0.001
ICD10	09 Disease of the circulatory system	I30-I52 Other forms of heart disease	I50 Heart failure	–	9	1.384	1.071	1.789	0.013
ICD10	09 Disease of the circulatory system	I70-I79 Diseases of arteries, arterioles and capillaries	I70 Atherosclerosis	–	3	1.501	1.351	1.666	<0.001
ICD10	11 Disease of the digestive system	K70-K77 Diseases of the liver	K76 Other diseases of the liver	Liver disease	2	1.943	1.752	2.156	<0.001
ICD10	13 Disease of the musculo-skeletal system and connective tissue	M05-M14 Inflammatory polyarthropaties	M12 Other specific arthropaties	Osteoarthritis, arthritis	3	1.692	1.250	2.292	0.001
ICD10	13 Disease of the musculo-skeletal system and connective tissue	M15-M19 Artrhosis	M19 Other arthrosis	History of joint replacement	1	3.726	1.360	10.250	0.002
ICD10	13 Disease of the musculo-skeletal system and connective tissue	M30-M36 Systemic connective tissue disorder	M35 Other systemic involvement of connective tissue	Connective tissue disease	1	1.387	1.215	1.530	<0.001
ICD10	18 Symptoms, signs, and abnormal clinical and laboratory findings	R25-R29 Symptoms and signs involving the nervous and musculoskeletal systems	R29.6 Tendency to fall, not elsewhere classified	–	25	2.211	1.774	2.754	<0.001
ICD10	18 Symptoms, signs, and abnormal clinical and laboratory findings	R50-R69 General symptoms and signs	R55 Syncope and collapse	–	1	2.170	1.077	4.371	0.030
ICD10	18 Symptoms, signs, and abnormal clinical and laboratory findings	R50-R69 General symptoms and signs	R65 Systemic Inflammatory Response Syndrome	Markers of acute infections (i.e., fever, leucocytosis, increased CRP)	5	1.462	1.221	1.751	<0.001
ICD10	19 Injury, poisoning and certain other consequences of external causes	–	T08-T14 Injuries to unspecified part of trunk, limb or body region	Fractures, lower limb amputation	5	1.390	1.048	1.844	0.022
ICD10	19 Injury, poisoning and certain other consequences of external causes	Y70-Y82 Medical devices associated with adverse incidents in diagnostic and therapeutic use	Y74.1 Therapeutic (nonsurgical) and rehabilitative devices	Devices, e.g., bed restraints, bedrails, handrails, lines, tubes, drains, etc	3	3.789	1.359	10.560	0.011
ICD10	20 External causes of morbidity and mortality	W00-X59 Other external causes of accidental injury	W19 Unspecified Falls	Recent fall; fall as presenting complaint	1	2.650	1.313	5.350	0.007
ICD10	21 Factors influencing health status and contact with health services	Z40-Z54 Persons encountering health services for specific procedures and health care	Z50 Care involving use of rehabilitation procedures	Involvement in rehabilitation	3	2.913	2.788	3.045	<0.001
ATC	A Alimentary tract and metabolism	–	A07 Antidiarrheals, intestinal antiinflammatory / antiinfective agents	–	2	1.874	1.324	2.651	<0.001
ATC	A Alimentary tract and metabolism	–	A10 Drugs used in diabetes	–	6	1.939	1.205	3.120	0.006
ATC	B Blood and blood forming organs	B01 Antithrombotic agents	B01AD Enzymes	i.e., Trombolytic agents	1	8.609	3.486	21.264	<0.001
ATC	L Antineoplastic and immunomodulating agents	–	L01 Antineoplastic agents	–	2	2.165	1.331	3.520	0.002
ATC	N Nervous System	–	N02 Analgesics	–	15	1.521	1.018	2.274	0.041
ATC	N Nervous System	–	N03 Antiepileptics	–	12	2.009	1.508	2.677	<0.001
ATC	N Nervous System	N05 Psycholeptics	N05A Antipsychotic	–	17	1.862	1.439	2.411	<0.001
ATC	N Nervous System	N05 Psycholeptics	N05B Anxiolytics	–	12	1.930	1.572	2.369	<0.001
ATC	N Nervous System	N05 Psycholeptics	N05C Hypnotics and sedatives	–	15	1.685	1.193	2.380	0.003
ATC	N Nervous System	N06 Psychoanaleptics	N06A Antidepressants	–	13	1.299	1.045	1.613	0.018
ATC	R Respiratory system	–	R05 Cough and cold preparation	–	1	5.949	1.725	20.521	0.005
**Excluded categories**									
ICF	b2 Sensory functions and pain	b280-b289 Pain	b280 Sensation of pain	Protective factor	1	0.022	0.018	0.028	<0.001
ICF	b4 Functions of the cardiovascular, haematological, immunological, and respiratory systems	b410-b429 Functions of the cardiovacular system	–	Overlap with I50 and I70	9	1.168	1.001	1.363	0.049
ICF	b4 Functions of the cardiovascular, haematological, immunological, and respiratory systems	b430-b439 Functions of the haematological and immunological system	b430 Haematological system functions	Overlap with D64 ICD-10	2	1.366	1.063	1.756	0.015
ICF	b4 Functions of the cardiovascular, haematological, immunological, and respiratory systems	b430-b439 Functions of the haematological anndimmunological system	b435 Immunological system function	Overlap with R65 of ICD-10	3	1.366	1.139	1.639	0.001
ICF	b5 Functions of the digestive, metabolic and endocrine systems	b540 General metabolic functions	b5401 Carbohydrate metabolism	Overlap with E14 of ICD-10	11	1.432	1.123	1.825	0.004
ICF	b5 Functions of the digestive, metabolic and endocrine systems	b545 Water, mineral and electrolyte balance functions	b5452 Electrolyte balance	Overlap with E86 and E87 of ICD-10	4	1.764	1.443	2.157	<0.001
ICD10	05 Mental and Behavioral disorders	F00-F09 Organic, includic symptomatic, mental disorders	F00 Dementia in Alzheimer disease	Protective factor	1	0.242	0.134	0.438	<0.001
ICD10	05 Mental and Behavioral disorders	F00-F09 Organic, includic symptomatic, mental disorders	F05 Delirium, not induced by alcohol and other psychoactive substances	Overlap with categories mapping onto b1 of ICF	26	3.310	2.616	4.186	<0.001
ICD10	05 Mental and Behavioral disorders	F00-F09 Organic, includic symptomatic, mental disorders	F06 Other mental disorders due to brain damage and dysfunction and to physical disease	Overlap with categories mapping onto b1 of ICF	10	2.080	1.008	4.292	0.047
ICD10	09 Disease of the circulatory system	K00-K93 Unspecified disease of the digestive system	–	Protective factor	1	0.541	0.343	0.852	0.003
ICD10	09 Disease of the circulatory system	M00-M99 Unspecified diseases of the muscoloskeletal system and connective tissue	–	Protective factor	3	0.199	0.046	0.853	0.030
ATC	A Alimentary tract and metabolism	A02 drugs for acid-related disorders	A02A Antacids	Protective factor	1	0.690	0.501	0.950	0.023
ATC	A Alimentary tract and metabolism	A04 Antiemetics	A04A Antiemetics and antinauseants	Protective factor	1	0.610	0.450	0.827	0.001
ATC	C Cardiovascular system	C03D Potassium-sparing agents	–	Protective factor	2	0.556	0.375	0.827	0.004
ATC	G Genito-urinary system and sex hormones	G04 Urologicals	G04C Drugs used in benign prostatic hypertrophy	Protective factor	1	0.348	0.131	0.922	0.034
ATC	M Muscolo-skeletal system	M03 Muscle relaxants	–	Protective factor	1	0.430	0.279	0.663	<0.001
ATC	R Respiratory system	R01 Nasal preparation	–	Protective factor	1	0.520	0.324	0.835	0.007
ATC	R Respiratory system	R03 Drugs for obustructive airway disease	R03B Other drugs for obstruct airway diseases, inhalants	Protective factor	2	0.660	0.511	0.851	0.001

CL, classification; Rec, records; OR, odds ratio; L, lower; CI, confidence interval; U, upper. For simplicity, the Roman numerals in ICD10 have been replaced by Arabic numerals.

After this process, the purified list included 53 significant risk factors corresponding to 328 records. Of those risk factors, ICF and ICD-10 yielded twenty-one risk factors each, whereas the remaining eleven were linked to ATC. The pooled OR values of the purified risk factors list ranged from 1.299 (N06A Antidepressants) to 8.633 (d420 Transferring oneself). This final step, reducing the number of risk factors from 71 to 53, added a further 1.3 reduction factor. Overall, the whole process from meta-analysis to post-meta-analysis refinement reduced the initial set of 1,111 records to a final set of 53 significant risk factors, i.e., a 21 times reduction of the initial number.

## 4 Discussion

This study aimed to provide a proof of concept that it may be feasible to reduce the reported heterogeneity in the description of fall risk factors and, subsequently, their number by adopting the standard terminology provided by international health classifications. We achieved this aim in four subsequent steps. In particular, we first performed a literature review to identify the fall risk factors among hospitalized adults. After the study selection and their methodological evaluation, we linked the found risk factors to the corresponding ICF, ICD, and ATC health classifications categories, obtaining the first relevant reduction of the number of health concepts representing the found risk factors. Following this step, we performed a meta-analysis on these risk categories, identifying the significant ones, thus further reducing the number of categories. The post-meta-analysis refinement from linking redundancies and protective risk factors allowed us to enlist a final set of 53 risk factors across the three classifications, achieving a remarkable 21-reduction factor from the initial number of records. These results provided the proof of concept regarding the feasibility and validity of the proposed methodology.

This proof-of-concept study aimed to reduce the heterogeneity of fall risk factors by adopting a standard terminology provided by international health classifications. Thus, the first step was reviewing the literature, as in previous works, although we used a different approach to the study selection of earlier studies. For instance, Deandrea et al. included only studies with a prospective design, a sample size greater than 200 subjects, and subjects experiencing one or more falls during follow-up as an outcome (12). Furthermore, they considered only risk factors assessed by at least three studies. Following this selection, they found only six significant risk factors for hospital falls from 22 selected studies. This number of found risk factors is approximately in the same range as the number of items of some of the most common risk prediction tools [i.e., Stratify, Hendrick fall risk model II, Morse Fall scale, and Conley scale (64)], whose use was not recommended by the National Institute of Health Care Excellence (NICE) guidelines in 2013 (9), given their insufficient sensitivity and specificity for accurate and reliable predictions. On the other hand, our approach allowed us to enlist a final much larger set of risk factors (i.e., 53 distal-level categories vs. 6), thus possibly providing a more extensive content coverage of the ‘fall risk for inpatients’ phenomenon. We achieved this goal by adopting less restrictive criteria for study inclusion than those adopted by Deandrea et al. (12). In particular: (a) we included any observational study design without any low limitation for sample rather than just prospective studies with sample size >200 subjects as Deandrea et al. (12); (b) furthermore, while Deandrea et al. (12) selected only studies with 80% or more patients aged 65 years or older, we included studies conducted on inpatients of 16 years or older and excluded studies devoted only to nursing home residents. Thus, the selected sample will likely better represent the hospital inpatient population.

In the phase of extrapolating the risk factors from the selected studies, the extreme heterogeneity of these risk factors was immediately evident in terms of variability of the type of language used to define them and the methods of quantifying them. Indeed, we found great variability regarding the language used to describe them (which was not always consistent) and the methods used to quantify them as a risk factor, as they were different between the studies (e.g., the age defined by unequal classes for numbers of years or beyond a specific age). This extreme heterogeneity made the linking work quite complex and strengthened the need to develop a common terminology.

The linking process with the WHO Health Classifications was the core of this novel proof-of-concept study. We employed a modified version of the standard linking techniques available for the ICF, with different degrees of difficulty depending on the classification used. Regarding the ICF and the ATC, the association between fall risk factors and categories was relatively straightforward, as each risk factor could be linked to one of the 2^nd^-level categories of the two classifications. On the other hand, the same process with the ICD-10 was more complex because this classification has very specific categories and subcategories. In contrast, some risk factors were somehow too generic. Furthermore, as already reported, several risk factors could be linked to categories of different classifications (e.g., mental functions vs. corresponding diagnoses).

Indeed, the main outcome of the whole linking procedure is the empirical demonstration that the risk of falling is a multidimensional construct (9, 11, 12), with likely interactions between its various biological, behavioral, environmental, and socioeconomic dimensions, as proposed by the World Health Organization’s risk factor model for falls in older age (65). The multidimensionality of the fall risk construct emerged even within the ICF classification. Within the latter, we were able to link risk factors to different aspects of functioning, such as functions, activities, environmental, and personal factors, which are likely to interact with one another.

Amongst the functions, we identified various aspects of impairments of the cognitive functions (i.e., consciousness, orientation, intellectual, attention, psychomotor, higher-level), with OR ranging from 2.57 to 4.76. Our results are in line with those of Deandrea et al. (12), although they identified only one variable related to cognitive impairment, as quantified with the Mini-Mental State Examination and with a lower OR (1.52). Besides, further systematic reviews (without meta-analysis) reported these aspects as significant in determining falls (15, 66, 67), as well as the NICE guidelines (9). We also identified categories related to sensory impairments with high OR, such as alteration of the seeing function (OR: 3.86), confirmed by Todd et al.’s report (15) and by the NICE guidelines (9). This risk factor was reported by Deandrea et al. (12) as non-significant within the nursing home setting, although impaired vision can prevent, for instance, seeing obstacles along the way, causing trips, or correctly perceiving the distance from a chair in the postural change from standing to sitting. At the same time, we also found high OR for risk factors (i.e., dizziness and vertigo) linked to impairments of hearing and vestibular functions. Deandrea et al. (12) also identified these impairments with significant OR (1.52), although only for the nursing home setting, as other already cited studies (15, 67). Amongst the functions, we also identified the impairment of defecation that may lead to slips, trips and falls, especially if characterized by urge continence in association with sensory, cognitive, and/or motor deficits (68).

We found other significant risk factors (OR range: 1.73–8.63) linked to motor functions and activities, such as impairments of muscle power, involuntary movement reactions, gait patterns, transfers, walking, and washing oneself. Indeed, an impairment of the cognitive, sensory, and/or motor functions is likely to lead to the most recognized risk factors for falling (69), such as transfers and washing oneself, which yielded the highest OR (8.63 and 6.16, respectively). These activities have already been described in the literature as exposing patients to a greater risk of falling (9, 13, 15, 66, 67). In particular, safe transfers require executing a series of subsequent steps (e.g., bringing the wheelchair to the bed, placing both brakes, etc.) that may be difficult to perform in case of concurrent sensory, cognitive, or motor impairments. Those impairments may easily lead to lower limb failure during standing with subsequent falls. On the other hand, washing oneself is associated with an increased risk of falling if coupled with water spillage on the floor, leading to a slip and subsequent loss of balance.

Among the ICF environmental factors, walking and mobility aids could be linked to one significant ICF environmental category (e120). This finding could be explained considering that the use of these aids is often seen in older patients with impaired gait who often assume psychotropic medications (70). Interestingly, this risk factor was reported by Todd et al. (15) and found to be significant by Deandrea et al. (12) only within the nursing home population. This is probably due to the larger scope of our literature review compared to the more restrictive one of Deandrea et al. (12), as mentioned previously. Within ICF’s environmental factors, we could also link up to 278 medication records to just one category (i.e., e110). However, considering the need to distinguish between classes of medications, we decided to link to e110 only the 18 records that were not specifically linkable to a drug class (e.g., ‘polypharmacotherapy’).

In contrast, we linked the remaining 260 records to specific categories of the ATC classification. In this way, we were able to find significant ORs for several classes of medications, such as alimentary tract and metabolism medications (i.e., antidiarrheal and drugs for diabetes), thrombolytic agents, antineoplastic agents, several classes of psychotropic medications (i.e., analgesics, antiepileptics, anxiolytics, hypnotic and sedatives, and antidepressants), and preparations for coughs and colds. Sedatives and antidepressants were also identified as prominent risk factors by Deandrea et al. (12) and by further authors in systematic reviews without meta-analysis (13, 15, 66). Given their interference with several body functions, these medications are likely associated with fall risk. For instance, psychotropic drugs are likely to have a direct impact on cognitive and motor functions (71). In contrast, antithrombotic agents (whose OR was the highest one within the various ATC classes) are likely to have an indirect impact, as these medications are used in acute stroke that, in turn, leads to sensory, cognitive, and motor impairments. Also, cough and cold preparations were associated with a high OR: this could be explained considering that these medications often contain codeine, which is an opiate known to interfere with cognitive functioning.

Amongst the personal factors, De Andrea et al. (12), in their systematic review and meta-analysis of risk factors in older people in nursing homes and hospitals, used an *ad hoc* statistical method for risk factors requiring a dose–response analysis (e.g., age), which allowed their comparison even when reported in different ways between studies. However, as we included more studies (36 vs. 22), we found significant heterogeneity in how age was reported between studies. In particular, in those where age was expressed as categories that could be homogenously dichotomized (e.g., in ≤70 or > 70 years), the OR was 1.25 (95%CI: 0.33–4.77). In contrast, when age was reported as a continuous variable, the pooled OR was 0.11 (95%CI: −0.06–0.27). In both cases, the pooled OR were not statistically significant. Our results also showed that both genders were not associated with a significantly increased risk of falls, as the OR was 1.11 (95%CI: 0.94–1.32) for males and 0.75 (95%CI: 0.48–1.15) for females.

We were able to find further significant fall risk factors, such as Parkinson’s disease, epilepsy, paraplegia, and tetraplegia, which were linked to the ICD-10 (OR range: 1.76–3.11). All these factors are classified as diseases of the nervous system that cause motor and balance impairment, which can easily be associated with an increased fall risk (10). We also found other pathologies linked to the ICD-10, like diabetes mellitus or malignant neoplasms, associated with significant risk factors (OR 1.43 and 1.62, respectively). Although these pathologies are traditionally considered risk factors by themselves, we should consider that they may increase fall risk indirectly by acting upon the various aspects of functioning described by ICF. For instance, patients with diabetes mellitus may suffer from diabetic neuropathy that is characterized by motor (i.e., b730) and sensitive impairments (b265). Also, patients with diabetes may suffer from visual function impairments (b210) (15). In the most extreme cases, a severe diabetic neuropathy associated with arterial vasculopathy (I70) may lead to limb amputations (T18-T14), which exposes the subject to a further increase in the risk of falling (67). Also, malignant tumors, especially in an advanced stage, can cause severe muscle weakness (b730), which in turn exposes the patient to the risk of falling (13). We also found an OR above 2 for a history of falls, which is confirmed to be one of the most frequently reported risk factors for inpatient falls (9, 13, 15, 66, 67). As discussed by De Andrea et al. (12), this is not a causal factor but probably a manifestation of real underlying causal agents, such as impaired balance.

The last step of this novel proof-of-concept approach was a qualitative refinement undertaken post-meta-analysis. The latter regarded: (1) the removal of one of the conceptually overlapping categories between ICF and ICD-10 and (2) the deletion of modifiable risk factors found to be protective. The latter choice was in line with the main goal of this work, which was focused on fall risk factors rather than on factors to prevent falls. Furthermore, it would make little sense to include protective risk factors that are either health conditions or aspects of functioning that could not be replicated (e.g., dementia) or would be unethical to replicate if not present (e.g., pain) or even medications (e.g., antacids, antiemetics, medication for benign prostatic hypertrophy) that may not be appropriate to administer to an individual outside of the recommended prescribing indications.

There are other published systematic reviews related to fall risk factors within the hospital (13, 66, 67) and/or the nursing home setting (15). Some of these reviews reported as significant risk factors anti-hypertensive drugs (13, 67) and diuretics usage [13; 15; 65], urinary incontinence (13, 15, 66), age (15, 67, 72), and having had a stroke (15, 67, 73). On the other hand, other studies did not report at all factors related to blood pressure functions (66), neither age nor stroke (13), and gave evidence of the use of diuretics (67) as a non-significant risk factor. Our results did not confirm the significance of several well-known fall risk factors such as those related to *b420 blood pressure functions* (i.e., hypertension, orthostatic hypotension and related medications), *b6 genitourinary and reproductive functions* (i.e., urinary incontinence, use of a urinary catheter, and renal insufficiency), to *personal factors* (i.e., age), and *I60-I69 cerebrovascular diseases* (i.e., stroke). Therefore, we excluded them from the final risk factor list.

These discrepancies between our results and those of the studies mentioned above should be viewed in light of the different methodologies applied. In particular, those studies included one literature report (15) and three systematic reviews (13, 66, 67), but none of them performed a meta-analysis. Indeed, those studies reported the significance of certain fall risk factors only according to the reports of single primary studies, without verifying the actual significance across the various studies by performing a quantitative synthesis of the results. On the other hand, the only study that conducted a meta-analysis (Deandrea et al.) reported only age as a significant hospital risk factor. In contrast, hypertension, orthostatic hypotension and related medications did not even meet the criteria to perform the meta-analysis. Furthermore, urinary incontinence and stroke resulted as non-significant risk factors within the meta-analysis conducted for the nursing home setting (12). In other words, with the exception of age, our results can be considered quite in line with those from the only study where a quantitative analysis (i.e., a meta-analysis) was performed (12).

This study has further limitations that should be highlighted. First, we included only articles published in English and Italian; thus, possibly relevant articles in different languages were not included in our literature review. Second, although we adopted some of the standards provided by the PRISMA statement, our literature review cannot be considered systematic. In particular, we included some primary studies, but not all of them were published before 2015. We also performed a literature search limited to four but not all available databases (e.g., PsychINFO and Web of Science were not included). Besides, we did not perform an accurate risk-of-bias assessment, including the publication bias. However, it should be pointed out that the units of analysis were the risk factors and not the studies, and therefore, it made little sense to investigate this bias. Third, as the outcome detection was implemented with different modalities across the selected studies, this issue might have influenced our results. Finally, the suboptimal quality of some of the included studies may have negatively affected some of the risk factors found.

Notwithstanding these limitations, this proof-of-concept study demonstrated that adopting a clear and consistent terminology derived from standardized international classifications may lead to a marked reduction and systematization of fall risk factors among hospitalized adults. Even though preventing in-hospital falls is a public health priority (7), current evidence suggests there are neither valid instruments for predicting fall risk factors (9) nor ultimate effective interventions to reduce falls for inpatients (74). Thus, the larger set of risk factors proposed here may be useful for critically appraising the content coverage of existing tools for predicting falls, developing better prediction tools, and, likely, novel therapeutic interventions to prevent falls in future research. Finally, once refined, the methodology adopted within this work may pave the way toward developing a standardized taxonomy of fall risk factors. This will undoubtedly improve the reporting of fall risk factors and the methodological quality of future studies exploring the assessment of risk factors and interventions to reduce in-hospital falls.

## Data availability statement

The raw data supporting the conclusions of this article will be made available by the authors, without undue reservation.

## Author contributions

FP: Conceptualization, Methodology, Supervision, Writing – original draft, Writing – review & editing. GV: Formal analysis, Investigation, Software, Writing – review & editing. GL: Supervision, Writing – review & editing. AN: Data curation, Formal analysis, Investigation, Software, Writing – review & editing. LP: Writing – original draft, Writing – review & editing. EB: Data curation, Writing – review & editing. SC: Conceptualization, Methodology, Writing – original draft, Writing – review & editing. VP: Data curation, Investigation, Writing – review & editing. EG: Conceptualization, Methodology, Supervision, Writing – review & editing.
