# Supplementary material for: A novel multistep approach to standardize the reported risk factors for in-hospital falls: a proof-of-concept study
Source: Front Public Health. 2024 Jun 12;12:1390185. doi: 10.3389/fpubh.2024.1390185 (PMC11199548; doi:10.3389/fpubh.2024.1390185)
Supplement: Supplementary file 1 [file Table_1.DOCX]

Supplementary Material

**Supplementary Table 1. Full search strings for each database**

| **Database** | **Filters** | **Search string** |
| --- | --- | --- |
| PubMed | Abstract, in the last 5 years, English, Italian, Aged: 65+ years | "accidental falls"[MeSH Terms] AND "risk factors"[MeSH Terms] AND "hospitalization"[MeSH Terms] |
| CINAHL | No time limits | TI accidental falls AND TI risk factors |
|  |  | AB falls in hospitals AND AB risk factors |
| EMBASE | Abstract, in the last 5 years, English, Italian, Aged: 65+ years | falls:ab,ti AND 'risk factor':ab,ti AND hospital:ab,ti |
| Scopus | LIMIT-TO TITLE-ABS-KEY (PUBYEAR, 2020) OR LIMIT-TO (PUBYEAR , 2019) OR LIMIT-TO (PUBYEAR , 2018) OR LIMIT-TO (PUBYEAR , 2017) OR LIMIT-TO (PUBYEAR , 2016) OR LIMIT-TO (PUBYEAR , 2015) | "fall risk factor*" AND hospital OR "acute care" AND adult OR elderly |

**Supplementary Table 2.** **Quality assessment of the case-control studies included in this review**

| **Study** | **Item1** | **Item2** | **Item3** | **Item4** | **Item5** | **Item6** | **Item7** | **Item8** | **Item9** | **Item10** | **Item11** | **Item12** | **Study quality** |
| --- | --- | --- | --- | --- | --- | --- | --- | --- | --- | --- | --- | --- | --- |
| Aryee et al. 2017 [26] | Yes | Yes | Yes | Yes | Yes | Yes | No | Yes | No | No | No | Yes | Fair |
| Chang et al. 2011 [29] | Yes | Yes | No | Yes | Yes | Yes | Yes | Yes | Yes | Yes | Yes | Yes | Good |
| Cho et al. 2020 [28] | Yes | Yes | No | Yes | Yes | Yes | No | Yes | Yes | Yes | No | Yes | Good |
| Cox et al. 2017 [30] | Yes | Yes | No | Yes | Yes | Yes | No | Yes | CD | CD | NR | Yes | Fair |
| Fehlberg et al. 2017 [32] | Yes | Yes | No | Yes | Yes | Yes | No | Yes | Yes | Yes | Yes | Yes | Good |
| Guzzo et al. 2015 [34] | Yes | Yes | No | yes | Yes | Yes | Yes | Yes | Yes | Yes | NR | Yes | Good |
| Ishibashi et al. 2020 [38] | Yes | Yes | Yes | Yes | Yes | Yes | Yes | CD | Yes | CD | No | Yes | Fair |
| Lucero et al. 2019 [44] | Yes | Yes | No | Yes | Yes | Yes | Yes | Yes | Yes | Yes | No | Yes | Good |
| Mamun & Lim, 2009 [46] | Yes | No | No | yes | yes | Yes | No | Yes | Yes | Yes | NR | Yes | Fair |
| Morishita et al. 2022 [48] | Yes | Yes | No | Yes | NR | Yes | No | Yes | Yes | Yes | NR | Yes | Good |
| Najafpour et al. 2019 [49] | Yes | Yes | No | Yes | Yes | No | NA | Yes | CD | CD | No | No | Poor |
| Nanda et al. 2011 [50] | Yes | No | No | No | no | Yes | yes | Yes | CD | Yes | NR | No | Poor |
| Noh et al. 2021 [51] | Yes | Yes | Yes | Yes | Yes | Yes | No | Yes | Yes | Yes | NR | Yes | Good |
| Oneil et al. 2018 [53] | Yes | Yes | Yes | Yes | Yes | Yes | Yes | Yes | Yes | Yes | NR | Yes | Good |
| Severo et al. 2018 [55] | Yes | Yes | No | Yes | No | Yes | No | Yes | Yes | Yes | No | Yes | Fair |
| Swartzell et al. 2013 [57] | Yes | Yes | No | Yes | Yes | Yes | Yes | Yes | Yes | CD | NR | No | Fair |
| Vela et al. 2018 [59] | Yes | Yes | No | Yes | Yes | Yes | No | Yes | No | CD | NR | Yes | Fair |
| Wedmann et al. 2019 [60] | Yes | Yes | No | Yes | No | Yes | NR | CD | Yes | Yes | No | Yes | Fair |
| Yip et et al. 2016 [61] | Yes | Yes | No | Yes | NR | Yes | Yes | CD | CD | CD | NR | Yes | Fair |

**Abbreviations:** CD, cannot determined; NR, not reported; NA, not applicable

**Notes:**

Item1. Was the research question or objective in this paper clearly stated and appropriate?

Item2. Was the study population clearly specified and defined?
Item3. Did the authors include a sample size justification?

Item4. Were controls selected or recruited from the same or similar population that gave rise to the cases (including the same timeframe)?

Item5. Were the definitions, inclusion and exclusion criteria, algorithms or processes used to identify or select cases and controls valid, reliable, and implemented consistently across all study participants?

Item6. Were the cases clearly defined and differentiated from controls?

Item7. If less than 100 percent of eligible cases and/or controls were selected for the study, were the cases and/or controls randomly selected from those eligible?

Item8. Was there use of concurrent controls?

Item9. Were the investigators able to confirm that the exposure/risk occurred prior to the development of the condition or event that defined a participant as a case?

Item10. Were the measures of exposure/risk clearly defined, valid, reliable, and implemented consistently (including the same time period) across all study participants?

Item11. Were the assessors of exposure/risk blinded to the case or control status of participants?

Item12. Were key potential confounding variables measured and adjusted statistically in the analyses? If matching was used, did the investigators account for matching during study analysis?

**Supplementary Table 3.** **Quality assessment of the cross-sectional and cohort studies included in this review**

| **Study** | **Item1** | **Item2** | **Item3** | **Item4** | **Item5** | **Item6** | **Item7** | **Item8** | **Item9** | **Item10** | **Item11** | **Item12** | **Item13** | **Item14** | **Study quality** |
| --- | --- | --- | --- | --- | --- | --- | --- | --- | --- | --- | --- | --- | --- | --- | --- |
| Akgün et al. 2022 [23] | Yes | Yes | Yes | Yes | No | CD | CD | No | CD | No | CD | CD | CD | No | Fair |
| al Tehewy et al. 2015 [24] | Yes | Yes | Yes | Yes | Yes | Yes | Yes | Yes | Yes | Yes | Yes | NR | Yes | Yes | Good |
| Aranda-Gallardo et al. 2017 [25] | Yes | Yes | NR | Yes | Yes | Yes | NR | NR | Yes | Yes | Yes | Yes | No | Yes | Good |
| Brand & Sundararajan, 2010 [27] | Yes | Yes | Yes | Yes | Yes | NR | CD | NA | Yes | No | Yes | No | Yes | Yes | Fair |
| Eglseer et al. 2020 [31] | Yes | Yes | NR | Yes | No | No | No | NA | Yes | No | Yes | NR | No | Yes | Poor |
| Forrest & Chen, 2016 [33] | Yes | Yes | Yes | Yes | Yes | Yes | CD | Yes | Yes | No | Yes | NR | Yes | Yes | Good |
| Hanger et al. 2014 [35] | Yes | Yes | Yes | NR | No | Yes | CD | No | No | No | Yes | NR | Yes | No | Poor |
| Hauer et al. 2020 [36] | Yes | Yes | NR | Yes | No | Yes | CD | NA | Yes | No | Yes | No | Yes | Yes | Fair |
| Hou et al. 2017 [37] | Yes | No | Yes | Yes | No | Yes | CD | Yes | Yes | No | Yes | NR | NR | Yes | Fair |
| Ishikuro et al. 2017 [39] | Yes | Yes | Yes | Yes | Yes | Yes | CD | Yes | Yes | No | Yes | NR | Yes | Yes | Good |
| Jung & Park, 2018 [40] | Yes | Yes | Yes | Yes | No | Yes | CD | NA | Yes | Yes | Yes | No | Yes | Yes | Good |
| Juraschek et al. 2019 [41] | Yes | Yes | No | CD | No | Yes | CD | Yes | CD | No | Yes | NR | Yes | Yes | Fair |
| Kim et al. 2019 [42] | Yes | Yes | CD | Yes | No | Yes | NR | Yes | Yes | No | Yes | No | Yes | Yes | Fair |
| Lackoff et al. 2020 [43] | Yes | Yes | Yes | Yes | No | Yes | Yes | Yes | Yes | No | Yes | No | Yes | Yes | Good |
| Magnuszewki et al. 2020 [45] | Yes | Yes | Yes | Yes | Yes | Yes | NR | Yes | Yes | No | Yes | NR | NR | Yes | Fair |
| Mazur et al. 2016 [47] | Yes | Yes | Yes | NR | CD | Yes | CD | Yes | CD | No | Yes | NR | Yes | Yes | Fair |
| Obayashi et al. 2013 [52] | Yes | Yes | Yes | Yes | Yes | Yes | CD | Yes | Yes | No | Yes | NR | Yes | Yes | Good |
| Pauley et al. 2006 [54] | Yes | Yes | Yes | Yes | No | Yes | CD | Yes | Yes | No | Yes | NR | Yes | Yes | Good |
| Sullivan & Harding, 2019 [56] | Yes | Yes | Yes | Yes | Yes | Yes | Yes | NA | Yes | No | Yes | No | Yes | Yes | Good |
| Toye et al. 2019 [58] | Yes | Yes | Yes | Yes | No | Yes | Yes | Yes | Yes | CD | Yes | No | NR | Yes | Good |
| Yu et al. 2010 [62] | Yes | Yes | Yes | Yes | No | Yes | CD | No | Yes | No | Yes | NR | Yes | Yes | Good |

**Abbreviations:** NR, not reported; CD, cannot determine; NA, not applicable

**Notes:**

Item1. Was the research question or objective in this paper clearly stated?

Item2. Was the study population clearly specified and defined?

Item3. Was the participation rate of eligible persons at least 50%?

Item4. Were all the subjects selected or recruited from the same or similar populations (including the same time period)? Were inclusion and exclusion criteria for being in the study prespecified and applied uniformly to all participants?

Item5. Was a sample size justification, power description, or variance and effect estimates provided?

Item6. For the analyses in this paper, were the exposure(s) of interest measured prior to the outcome(s) being measured?

Item7. Was the timeframe sufficient so that one could reasonably expect to see an association between exposure and outcome if it existed?

Item8. For exposures that can vary in amount or level, did the study examine different levels of the exposure as related to the outcome (e.g., categories of exposure, or exposure measured as continuous variable)?

Item9. Were the exposure measures (independent variables) clearly defined, valid, reliable, and implemented consistently across all study participants?

Item10. Was the exposure(s) assessed more than once over time?

Item11. Were the outcome measures (dependent variables) clearly defined, valid, reliable, and implemented consistently across all study participants?

Item12. Were the outcome assessors blinded to the exposure status of participants?

Item13. Was loss to follow-up after baseline 20% or less?

Item14. Were key potential confounding variables measured and adjusted statistically for their impact on the relationship between exposure(s) and outcome(s)?

**Supplementary Material 4. Non-significant risk factors linked to the International Classification of Functioning, Disability, and Health (ICF)**

| **Risk Factor** | | | | **N records** | **OR** | **Lower 95% CI** | **Upper 95% CI** | **p-value** |
| --- | --- | --- | --- | --- | --- | --- | --- | --- |
|  |  | b130 Energy and drive functions | | 1 | 1.020 | 0.198 | 5.258 | 0.975 |
|  |  | b134 Sleep functions | | 4 | 1.623 | 0.825 | 3.193 | 0.084 |
|  |  | b144 Memory functions | | 1 | 0.330 | 0.039 | 2.818 | 0.311 |
|  |  | b152 Emotional functions | | 7 | 1.846 | 0.799 | 4.263 | 0.151 |
|  |  | b156 Perceptual functions | | 2 | 1.028 | 0.178 | 5.946 | 0.976 |
|  |  | b167 Mental functions of language | | 2 | 1.328 | 0.334 | 5.284 | 0.687 |
|  |  | b410 Heart functions | | Not metanalyzable | | | | |
|  |  | b420 Blood pressure functions | | 9 | 1.108 | 0.914 | 1.342 | 0.299 |
|  | b510-b539 Functions related to the digestive system | | | 16 | 1.226 | 0.746 | 2.014 | 0.421 |
|  |  | b515 Digestive functions | | Not metanalyzable | | | | |
|  |  |  | b5158 Digestive functions, other specified | Not metanalyzable | | | | |
|  |  | b530 Weight maintenance functions | | 15 | 1.144 | 0.687 | 1.906 | 0.605 |
|  |  |  | b5403 Fat metabolism | Not metanalyzable | | | | |
|  |  |  | b5451 Mineral balance | Not metanalyzable | | | | |
|  |  | b550 Thermoregulatory functions | | 2 | 1.250 | 0.335 | 4.668 | 0.739 |
|  |  | b598 Functions of the digestive, metabolic and endocrine systems, other specified | | Not metanalyzable | | | | |
| **b6 genitourinary and reproductive functions** | | | | 25 | 1.560 | 1.000 | 2.433 | 0.050 |
|  | b610-b639 Urinary functions | | | 25 | 1.560 | 1.000 | 2.433 | 0.050 |
|  |  | b610 Urinary excretory functions | | 4 | 1.106 | 0.966 | 1.266 | 0.144 |
|  |  | b620 Urination functions | | 21 | 1.625 | 0.893 | 2.958 | 0.112 |
|  | b710-b729 Functions of the joints and bones | | | Not computable | | | | |
|  |  | b729 Functions of the joints and bones, other specified and unspecified | | Not computable | | | | |
| **b8 Functions of the skin and related structures** | | | | Not computable | | | | |
|  | b810-b849 Functions of the skin | | | Not computable | | | | |
|  |  | b810 Protective function of the skin | | Not computable | | | | |
|  |  | d410 Changing basic body position | | 2 | 1.971 | 0.723 | 5.373 | 0.185 |
|  |  | d530 Toileting | | 2 | 3.450 | 0.509 | 23.38 | 0.205 |
|  |  | d550 Eating | | 1 | 1.070 | 0.777 | 1.474 | 0.679 |
| **e1 Products and technology** | | | | 41 | 4.759 | 0.677 | 33.46 | 0.117 |
|  |  | e115 Products and technology for personal use in daily living | | 15 | 1.331 | 0.869 | 2.039 | 0.189 |
|  |  |  | e1150 General products and technology for personal use in daily living | 11 | 1.127 | 0.753 | 1.687 | 0.561 |
|  |  |  | e1151 Assistive products and technology for personal use in daily living | 4 | 2.170 | 0.566 | 8.313 | 0.258 |
|  |  | e125 Products and technology for communication | | 1 | 1.170 | 0.866 | 1.581 | 0.307 |
|  |  |  | e1251 Assistive products and technology for communication | 1 | 1.170 | 0.866 | 1.581 | 0.307 |
|  |  | e150 Design, construction and building products and technology of buildings for public use | | 1 | 1.380 | 0.854 | 2.230 | 0.188 |
|  |  | e155 Design, construction and building products and technology of buildings for private use | | 2 | 1.046 | 0.398 | 2.746 | 0.927 |
|  |  |  | e1553 Design, construction and building products and technology for physical safety of persons in buildings for private use | 2 | 1.046 | 0.398 | 2.746 | 0.927 |
|  |  |  | e1558 Design, construction and building products and technology of buildings for private use, other specified | Not computable | | | | |
| **e3 Support and relationships** | | | | 6 | 1.036 | 0.620 | 1.730 | 0.894 |
|  |  | e310 Immediate family | | 6 | 1.036 | 0.620 | 1.730 | 0.894 |
| **e5 Services, systems and policies** | | | | 35 | 1.166 | 0.865 | 1.573 | 0.312 |
|  |  | e580 health services, systems and policies | | 35 | 1.166 | 0.865 | 1.573 | 0.312 |
|  |  | hc.misc | | 1 | 1.830 | 0.805 | 4.158 | 0.150 |
| **pf Person Factors** | | | | Not meta-analyzable | | | | |
|  |  | Age | | Not meta-analyzable | | | | |
|  |  | Gender | | Not meta-analyzable | | | | |
|  |  | Ethnicity / Nationality | | Not meta-analyzable | | | | |
|  |  | Domicile | | Not meta-analyzable | | | | |
|  |  | Socio-economic | | Not meta-analyzable | | | | |
|  |  | Miscellaneous | | Not meta-analyzable | | | | |

**Abbreviations:** N, number; OR, odds ratio, CI, confidence interval.

**Notes:**

Risk factors reported as “not computable” represent those who, due to mathematical issues (e.g.studies reporting a null number of exposed cases or else) and the absence of refined results as unadjusted/adjusted OR, cannot be computed.

Risk factors reported as “not meta-analyzable” means that the risk factors are found in the literature but, due to the way results are reported, they should be included in the assessment of risk factors but cannot be analyzed

**Supplementary Table 5**. **Non-significant risk factors linked to the International Classification of Diseases (ICD10)**

| **Risk Factor** | | | **N records** | **OR** | **Lower 95% CI** | **Upper 95% CI** | **p-value** |
| --- | --- | --- | --- | --- | --- | --- | --- |
| **01 Certain infections and parasitic diseases** | | | N/A | | | | |
|  | B20-B24 Human Immunodeficiency virus [HIV] disease | | N/A | | | | |
|  |  | B24 Unspecified human immunodeficiency virus [HIV] disease | N/A | | | | |
|  |  | C81-C96 Malignant neoplasms, stated or presumed to be primary, of lymphoid, haematopoietic and related tissue | 3 | 1.618 | 0.677 | 3.871 | 0.279 |
|  | E40-E46 Malnutrition | | 15 | 1.144 | 0.687 | 1.906 | 0.605 |
|  |  | E46 Unspecified protein-energy malnutrition | 15 | 1.144 | 0.687 | 1.906 | 0.605 |
|  | E50-E64 Deficiency of other B group vitamins | | Not metanalyzable | | | | |
|  |  | E53 Dificiency of other B group vitamins | Not metanalyzable | | | | |
|  |  | E78 Disorders of lipoprotein metabolism and other lipidaemias | Not metanalyzable | | | | |
|  |  | F03 Unspecified dementia | 13 | 1.747 | 0.950 | 3.210 | 0.073 |
|  | F10-F19 Mental and behavioural disorders due to psychoactive substance use | | 4 | 1.389 | 0.551 | 3.504 | 0.486 |
|  |  | F10 Mental and behavioral disorders due to use of alcohol | 3 | 1.527 | 0.444 | 5.248 | 0.501 |
|  |  | F19 Mental and behavioural disorders due to multiple drug use and use of other psychoactive substances | 1 | 1.020 | 0.292 | 3.564 | 0.975 |
|  | F20-F29 Schizophrenia, schizotypical and delusional disorders | | 3 | 0.659 | 0.119 | 3.637 | 0.632 |
|  |  | F29 Unspecified nonorganic psychosis | 3 | 0.659 | 0.119 | 3.637 | 0.632 |
|  | F30-F39 Mood [affective disorders] | | 6 | 1.726 | 0.696 | 4.280 | 0.239 |
|  |  | F38 Other mood [affective] disorders | 6 | 1.726 | 0.696 | 4.280 | 0.239 |
|  |  | G47 Sleep disorders | 3 | 1.376 | 0.768 | 2.464 | 0.284 |
|  | G60-G64 Polineuropathies and other disorders of the peripheral nervous system | | 1 | 0.800 | 0.361 | 1.771 | 0.582 |
|  |  | G64 Other disorders of the peripheral nervous system | 1 | 0.800 | 0.361 | 1.771 | 0.582 |
|  | G89-G99 Other disorders of the nervous system | | 4 | 0.488 | 0.135 | 1.767 | 0.274 |
|  |  | G95 Other and unspecified diseases of spinal cord | 2 | 0.567 | 0.054 | 6.013 | 0.638 |
|  |  | 696 Other disorders of central nervous system | 2 | 0.401 | 0.070 | 2.291 | 0.304 |
| **07 Diseases of the eye and adnexa** | | | 3 | 0.325 | 0.070 | 1.507 | 0.151 |
|  | H25-H28 Disorders of the lens | | 1 | 0.999 | 0.199 | 5.028 | 0.999 |
|  | H26 Other cataract | | 1 | 0.999 | 0.199 | 5.028 | 0.999 |
|  | H40-H42 Glaucoma | | 1 | 0.500 | 0.045 | 5.568 | 0.571 |
|  | H40 Glaucoma | | 1 | 0.500 | 0.045 | 5.568 | 0.571 |
|  | I10-I15 Hypertensive diseases | | 6 | 1.116 | 0.885 | 1.407 | 0.353 |
|  | I10 Essential (primary) hypertension | | 6 | 1.116 | 0.885 | 1.407 | 0.353 |
|  | I20-I25 Ischaemic heart disease | | 5 | 1.033 | 0.961 | 1.110 | 0.377 |
|  |  | I24 Other acute ischaemic heart disease | 3 | 1.030 | 0.958 | 1.109 | 0.423 |
|  |  | I25 Chronic heart disease | 2 | 1.095 | 0.765 | 1.566 | 0.621 |
|  |  | I48 Atrial fibrillation and flutter | 1 | 1.003 | 0.610 | 1.647 | 0.992 |
|  | I60-I69 Cerebrovascular Diseases | | 11 | 1.199 | 0.712 | 2.020 | 0.494 |
|  |  | I63 Cerebral infarction | 1 | 1.241 | 0.889 | 1.732 | 0.206 |
|  |  | I64 Stroke, not specified as haemorrhage or infarction | 10 | 1.191 | 0.670 | 2.118 | 0.552 |
|  | I95-I99 Other and specified disorders of the circulatory system | | 3 | 1.205 | 0.886 | 1.639 | 0.235 |
|  |  | I95 Hypotension | 3 | 1.205 | 0.886 | 1.639 | 0.235 |
|  |  | I951 Orthostatic hypotension | 1 | 1.275 | 0.704 | 2.308 | 0.431 |
|  | I00-I99 Unspecified disease of the circulatory system | | 2 | 0.720 | 0.487 | 1.063 | 0.099 |
| **10 Diseases of the respiratory system** | | | 1 | 1.206 | N/A | N/A | N/A |
|  | J40-J47 Chronic lower respiratory disease | | 1 | 1.206 | N/A | N/A | N/A |
| **11 Diseases of the digestive system** | | | 4 | 1.317 | 0.907 | 1.914 | 0.148 |
| **12 Diseases of the skin and subcutaneous tissue** | | | Not computable | | | | |
|  | L80-L89 Other disorders of the skin and subcutaneous tissue | | Not computable | | | | |
|  |  | L89 Decubitus ulcer and pressure area | Not computable | | | | |
| **13 Diseases of the muscoloskeletal system and connective tissue** | | | 11 | 1.022 | 0.492 | 2.123 | 0.953 |
|  | M80-M94 Osteopathies and chondropaties | | 3 | 1.929 | 0.828 | 4.492 | 0.128 |
|  |  | M81 Osteoporosis without pathological fracture | 3 | 1.929 | 0.828 | 4.492 | 0.128 |
| **14 Diseases of the genitourinary system** | | | 7 | 1.244 | 0.822 | 1.883 | 0.303 |
|  | N17-N19 Renal failure | | 6 | 1.326 | 0.855 | 2.057 | 0.208 |
|  |  | N18 chronic renal failure | 4 | 1.500 | 0.844 | 2.664 | 0.167 |
|  |  | N19 Unspecified kidney failure | 2 | 1.112 | 0.964 | 1.283 | 0.146 |
|  | N00-N99 Unspecified disease of the genitourinary system | | 1 | 0.848 | 0.512 | 1.404 | 0.521 |
|  | R47-R49 Symptoms and signs involving speech and voice | | 2 | 1.328 | 0.334 | 5.284 | 0.687 |
|  |  | R47 Speech disturbances not classified elsewhere | 2 | 1.328 | 0.334 | 5.284 | 0.687 |
|  | R70-R79 Abnormal findings on examination of blood, without diagnosis | | Not metanalyzable | | | | |
|  |  | R74 Abnormal serum enzyme levels | Not metanalyzable | | | | |
|  |  | R79 Other abnormal findings of blood chemistry | Not metanalyzable | | | | |
|  | S00-S09 Injuries to the head | | 1 | 1.641 | 0.550 | 1.250 | 0.080 |
|  | T00-T07 Injuries involving multiple body regions, not elsewhere classified | | 1 | 0.343 | 0.080 | 0.490 | 0.281 |
|  | S00-T98 Unspecified injury, poisoning and certain consequences of external injuries | | 2 | 1.264 | 0.915 | 1.747 | 0.155 |
| **20 External causes of morbidity and mortality** | | | 14 | 1.528 | 0.972 | 2.401 | 0.066 |
|  | Y70-Y82 Medical devices associated with adverse incidents in diagnostic and therapeutic use | | 13 | 1.447 | 0.901 | 2.322 | 0.126 |
|  |  | Y74 General hospital and personal-use devices associated with adverse incidents | 13 | 1.447 | 0.901 | 2.322 | 0.126 |
|  |  | Y74.8 Miscellaneous devices, not elsewhere classified | 10 | 1.085 | 0.767 | 1.536 | 0.645 |
| **21 Factors influencing health status and contact with health services** | | | 42 | 1.429 | 0.89 | 2.295 | 0.140 |
|  | Z00-Z13 Persons encountering health services for examination and investigation | | 1 | 1.075 | 0.240 | 4.809 | 0.925 |
|  |  | Z04 Examination and observation for other reasons | 1 | 1.075 | 0.240 | 4.809 | 0.925 |
|  | Z40-Z54 Persons encountering health services for specific procedures and health care | | 29 | 1.529 | 0.860 | 2.720 | 0.148 |
|  |  | Z47 Other orthopaedic follow-up care | 1 | 0.657 | 0.258 | 1.674 | 0.378 |
|  |  | Z48 Other surgical follow-up care | 5 | 2.062 | 0.491 | 8.667 | 0.323 |
|  |  | Z48.0 Encounter for attention to dressings, sutures and drains | 1 | 1.600 | 0.523 | 4.891 | 0.410 |
|  |  | Z51 Other medical care | 15 | 1.414 | 0.590 | 3.391 | 0.438 |
|  |  | Z51.8 Other specified medical care | 3 | 1.193 | 0.832 | 1.711 | 0.338 |
|  |  | Z54 Convalescence | 5 | 1.197 | 0.449 | 3.191 | 0.719 |
|  |  | Z54.0 Convalescence following surgery | 5 | 1.197 | 0.449 | 3.191 | 0.719 |
|  | Z55-Z65 Persons with potential health hazards related to socioeconomic and psychosocial circumstances | | 2 | 0.983 | 0.648 | 1.492 | 0.937 |
|  |  | Z58 Problems related to physical environment | 2 | 0.983 | 0.648 | 1.492 | 0.937 |
|  | Z80-Z99 Persons with potential health hazards related to family and personal history and certain conditions influencing health status | | 3 | 1.324 | 0.276 | 6.361 | 0.726 |
|  |  | Z87 Personal history of other diseases and conditions | 4 | 1.200 | 0.800 | 1.800 | 0.378 |
|  |  | Z92 Personal history of medical treatment | 3 | 1.324 | 0.276 | 6.361 | 0.726 |
|  |  | Z94 Transplanted organ and tissue status | 3 | 1.342 | 0.657 | 2.741 | 0.419 |
|  |  | Z94.8 Other transplanted organ and tissue status | 3 | 1.342 | 0.657 | 2.741 | 0.419 |

**Abbreviations:** N, number; OR, odds ratio, CI, confidence interval, N/A, not applicable

**Notes:**

Risk factors reported as “not computable” represent those who, due to mathematical issues (e.g.studies reporting a null number of exposed cases or else) and the absence of refined results as unadjusted/adjusted OR, cannot be computed.

Risk factors reported as “not metanalyzable” means that the risk factors are found in the literature but, due to the way results are reported, they should be included in the assessment of risk factors but cannot be analyzed

Risk factors labeled as “not applicable” is reported when no numerical values are reported in the literature but risk factors are highlighted.

**Supplemental Table 6. Non-significant risk factors linked to the Anatomical Therapeutic Chemical Classification (ATC)**

| **Risk Factor** | | | **N records** | **OR** | **Lower 95% CI** | **Upper 95% CI** | **p-value** |
| --- | --- | --- | --- | --- | --- | --- | --- |
| **A Alimentary tract and metabolism** | | | 23 | 1.144 | 0.928 | 1.41 | 0.207 |
|  | A02 drugs for acid-related disorders | | 3 | 0.824 | 0.621 | 1.093 | 0.180 |
|  |  | A02B Drugs for peptic ulcer and gastro-oesophageal reflux disease | 2 | 0.91 | 0.604 | 1.37 | 0.651 |
|  | A03 Drugs for functional gastrointestinal disorder | | 2 | 0.903 | 0.495 | 1.648 | 0.740 |
|  | A06 Drugs for constipation | | 4 | 1.049 | 0.718 | 1.532 | 0.806 |
|  |  | A10A Insulins and analogues | 1 | 1.330 | 0.969 | 1.826 | 0.078 |
|  | A11 Vitamins | | 1 | 0.68 | 0.409 | 1.145 | 0.148 |
|  | A12 Mineral supplements | | Not metanalyzable | | | | |
|  |  | A12B Potassium | Not metanalyzable | | | | |
|  |  | A12A Calcium | Not metanalyzable | | | | |
|  | AX Unspecified Alimentary tract and metabolism | | 2 | 0.965 | 0.627 | 1.485 | 0.871 |
| **B Blood and blood forming organs** | | | 10 | 1.387 | 0.946 | 2.033 | 0.094 |
|  | B01 Antithrombotic agents | | 10 | 1.387 | 0.946 | 2.033 | 0.094 |
|  |  | B01A Antithrombotic agents | 9 | 1.155 | 0.841 | 1.588 | 0.373 |
|  | B03 Antianemic preparations | | Not metanalyzable | | | | |
| **C Cardiovascular system** | | | 56 | 0.946 | 0.847 | 1.056 | 0.320 |
|  | C01 Cardiac therapy | | 16 | 1.002 | 0.768 | 1.307 | 0.990 |
|  |  | C01B Antiarrhhytmics, Class I and III | 5 | 1.076 | 0.487 | 2.376 | 0.856 |
|  |  | C01A Cardiac glycosides | 3 | 0.795 | 0.284 | 2.225 | 0.662 |
|  |  | C01C Cardiac stimulants excl. Cardiac glycosides | 1 | 0.800 | 0.470 | 1.360 | 0.410 |
|  |  | C01D Vasodilators used in cardiac disease | 7 | 1.008 | 0.762 | 1.334 | 0.955 |
|  | C02 Antihypertensive | | 10 | 0.992 | 0.615 | 1.600 | 0.974 |
|  |  | C02A Antiadrenergic agents, centrally acting | 2 | 1.018 | 0.241 | 4.300 | 0.981 |
|  |  | C02C Antiadrenergic agents, peripherally acting | 3 | 0.919 | 0.656 | 1.287 | 0.622 |
|  | C03 Diuretics | | 11 | 0.899 | 0.734 | 1.101 | 0.303 |
|  |  | C03A Low-celiling diuretics, thiazides | 1 | 1.390 | 0.791 | 2.444 | 0.253 |
|  |  | C03C High-ceiling diuretics | 1 | 1.270 | 0.914 | 1.764 | 0.154 |
|  | C07 Beta Blocking agents | | 5 | 0.919 | 0.764 | 1.104 | 0.367 |
|  | C08 Calcium Channel blockers | | 4 | 0.862 | 0.707 | 1.051 | 0.142 |
|  | C09 Agents acting on the renin-angiotensin system | | 8 | 0.923 | 0.735 | 1.158 | 0.487 |
|  | C10 Lipid modifying agents | | 2 | 1.155 | 0.591 | 2.256 | 0.674 |
|  | CX Unspecified Cardiovascular system | | 1 | 0.740 | 0.520 | 1.052 | 0.094 |
| **G Genito-urinary system and sex hormones** | | | 3 | 0.900 | 0.318 | 2.546 | 0.843 |
|  | G03 Sex hormones | | 1 | 1.210 | 0.525 | 2.786 | 0.654 |
|  | G04 Urologicals | | 2 | 0.813 | 0.126 | 5.243 | 0.828 |
|  |  | G04B Urologicals | 1 | 2.36 | 0.479 | 11.631 | 0.291 |
|  |  | G04BD Drugs for urinary frequency and incontinence | 1 | 2.36 | 0.479 | 11.631 | 0.291 |
| **H Systemic Hormonal preparations** | | | 4 | 1.054 | 0.843 | 1.318 | 0.642 |
|  | H01 Pituitary and hypothalamic hormones and analogues | | Not metanalyzable | | | | |
|  | H01A Pituitary and hypothalamic hormones and analogues | | Not metanalyzable | | | | |
|  | H01AB Thyrothropine | | Not metanalyzable | | | | |
|  | H02 Corticosteroid for systemic use | | 2 | 0.894 | 0.621 | 1.288 | 0.549 |
|  | H03 Thyroid therapy | | 1 | 0.934 | 0.417 | 2.095 | 0.869 |
|  |  | H03A Thyroid preparation | 1 | 0.934 | 0.417 | 2.095 | 0.869 |
|  |  | H03B Antithyroid preparation | Not computable | | | | |
|  | HX Unspecified Systemic Hormonal preparations | | 1 | 1.200 | 0.887 | 1.623 | 0.236 |
| **L Antineoplastic and immunomodulating agents** | | | 5 | 1.475 | 0.863 | 2.521 | 0.155 |
|  | L04 Immunsuppressant | | 3 | 1.256 | 0.575 | 2.744 | 0.568 |
| **M Muscolo-skeletal system** | | | 7 | 0.887 | 0.65 | 1.211 | 0.451 |
|  | M01 Antiinflammatory and antirheumatic products | | 6 | 1.039 | 0.858 | 1.257 | 0.697 |
|  |  | N02A Opioids | 11 | 1.528 | 0.948 | 2.461 | 0.081 |
|  | N04 Antiparkinson drugs | | 6 | 1.527 | 0.566 | 4.123 | 0.404 |
|  |  | N04B Antiparkinson drugs | 6 | 1.527 | 0.566 | 4.123 | 0.404 |
|  |  | N06D Anti-dementia drugs | 5 | 1.786 | 0.461 | 6.923 | 0.402 |
|  | N07 Other Nervous System drugs | | Not computable | | | | |
|  |  | N07C Antivertigo preparation | Not computable | | | | |
|  | NX Unspecified Nervous System | | 5 | 1.031 | 0.227 | 4.687 | 0.969 |
| **R Respiratory system** | | | 14 | 0.886 | 0.696 | 1.127 | 0.323 |
|  |  | R03A Adrenergic, inhalants | 3 | 0.950 | 0.729 | 1.237 | 0.701 |
|  |  | R03D Other systemic drugs for obstructive airway disease | 1 | 0.841 | 0.431 | 1.639 | 0.611 |
|  | R06 Antihistamines for systemic use | | 4 | 1.174 | 0.551 | 2.502 | 0.677 |
|  | RX Unspecified Respiratory System | | 2 | 0.745 | 0.445 | 1.245 | 0.261 |

**Abbreviations:** N, number; OR, odds ratio, CI, confidence interval.

**Notes:**

Risk factors reported as “not computable” represent those who, due to mathematical issues (e.g.studies reporting a null number of exposed cases or else) and the absence of refined results as unadjusted/adjusted OR, cannot be computed.

Risk factors reported as “not metanalyzable” means that the risk factors are found in the literature but, due to the way results are reported, they should be included in the assessment of risk factors but cannot be analyzed
